# Search for supersymmetry in hadronic final states with missing transverse energy using the variables *α*_T_ and b-quark multiplicity in pp collisions at $\sqrt{s} = 8\ \mathrm{TeV}$

**DOI:** 10.1140/epjc/s10052-013-2568-6

**Published:** 2013-09-18

**Authors:** The CMS Collaboration, S. Chatrchyan, V. Khachatryan, A. M. Sirunyan, A. Tumasyan, W. Adam, T. Bergauer, M. Dragicevic, J. Erö, C. Fabjan, M. Friedl, R. Frühwirth, V. M. Ghete, N. Hörmann, J. Hrubec, M. Jeitler, W. Kiesenhofer, V. Knünz, M. Krammer, I. Krätschmer, D. Liko, I. Mikulec, D. Rabady, B. Rahbaran, C. Rohringer, H. Rohringer, R. Schöfbeck, J. Strauss, A. Taurok, W. Treberer-treberspurg, W. Waltenberger, C.-E. Wulz, V. Mossolov, N. Shumeiko, J. Suarez Gonzalez, S. Alderweireldt, M. Bansal, S. Bansal, T. Cornelis, E. A. De Wolf, X. Janssen, A. Knutsson, S. Luyckx, L. Mucibello, S. Ochesanu, B. Roland, R. Rougny, H. Van Haevermaet, P. Van Mechelen, N. Van Remortel, A. Van Spilbeeck, F. Blekman, S. Blyweert, J. D’Hondt, R. Gonzalez Suarez, A. Kalogeropoulos, J. Keaveney, M. Maes, A. Olbrechts, S. Tavernier, W. Van Doninck, P. Van Mulders, G. P. Van Onsem, I. Villella, B. Clerbaux, G. De Lentdecker, V. Dero, A. P. R. Gay, T. Hreus, A. Léonard, P. E. Marage, A. Mohammadi, T. Reis, L. Thomas, C. Vander Velde, P. Vanlaer, J. Wang, V. Adler, K. Beernaert, L. Benucci, A. Cimmino, S. Costantini, G. Garcia, M. Grunewald, B. Klein, J. Lellouch, A. Marinov, J. Mccartin, A. A. Ocampo Rios, D. Ryckbosch, M. Sigamani, N. Strobbe, F. Thyssen, M. Tytgat, S. Walsh, E. Yazgan, N. Zaganidis, S. Basegmez, G. Bruno, R. Castello, L. Ceard, C. Delaere, T. du Pree, D. Favart, L. Forthomme, A. Giammanco, J. Hollar, V. Lemaitre, J. Liao, O. Militaru, C. Nuttens, D. Pagano, A. Pin, K. Piotrzkowski, A. Popov, M. Selvaggi, J. M. Vizan Garcia, N. Beliy, T. Caebergs, E. Daubie, G. H. Hammad, G. A. Alves, M. Correa Martins Junior, T. Martins, M. E. Pol, M. H. G. Souza, W. L. Aldá Júnior, W. Carvalho, J. Chinellato, A. Custódio, E. M. Da Costa, D. De Jesus Damiao, C. De Oliveira Martins, S. Fonseca De Souza, H. Malbouisson, M. Malek, D. Matos Figueiredo, L. Mundim, H. Nogima, W. L. Prado Da Silva, A. Santoro, L. Soares Jorge, A. Sznajder, E. J. Tonelli Manganote, A. Vilela Pereira, T. S. Anjos, C. A. Bernardes, F. A. Dias, T. R. Fernandez Perez Tomei, E. M. Gregores, C. Lagana, F. Marinho, P. G. Mercadante, S. F. Novaes, Sandra S. Padula, V. Genchev, P. Iaydjiev, S. Piperov, M. Rodozov, S. Stoykova, G. Sultanov, V. Tcholakov, R. Trayanov, M. Vutova, A. Dimitrov, R. Hadjiiska, V. Kozhuharov, L. Litov, B. Pavlov, P. Petkov, J. G. Bian, G. M. Chen, H. S. Chen, C. H. Jiang, D. Liang, S. Liang, X. Meng, J. Tao, J. Wang, X. Wang, Z. Wang, H. Xiao, M. Xu, C. Asawatangtrakuldee, Y. Ban, Y. Guo, W. Li, S. Liu, Y. Mao, S. J. Qian, H. Teng, D. Wang, L. Zhang, W. Zou, C. Avila, C. A. Carrillo Montoya, J. P. Gomez, B. Gomez Moreno, J. C. Sanabria, N. Godinovic, D. Lelas, R. Plestina, D. Polic, I. Puljak, Z. Antunovic, M. Kovac, V. Brigljevic, S. Duric, K. Kadija, J. Luetic, D. Mekterovic, S. Morovic, L. Tikvica, A. Attikis, G. Mavromanolakis, J. Mousa, C. Nicolaou, F. Ptochos, P. A. Razis, M. Finger, M. Finger, Y. Assran, A. Ellithi Kamel, A. M. Kuotb Awad, M. A. Mahmoud, A. Radi, M. Kadastik, M. Müntel, M. Murumaa, M. Raidal, L. Rebane, A. Tiko, P. Eerola, G. Fedi, M. Voutilainen, J. Härkönen, V. Karimäki, R. Kinnunen, M. J. Kortelainen, T. Lampén, K. Lassila-Perini, S. Lehti, T. Lindén, P. Luukka, T. Mäenpää, T. Peltola, E. Tuominen, J. Tuominiemi, E. Tuovinen, L. Wendland, A. Korpela, T. Tuuva, M. Besancon, S. Choudhury, F. Couderc, M. Dejardin, D. Denegri, B. Fabbro, J. L. Faure, F. Ferri, S. Ganjour, A. Givernaud, P. Gras, G. Hamel de Monchenault, P. Jarry, E. Locci, J. Malcles, L. Millischer, A. Nayak, J. Rander, A. Rosowsky, M. Titov, S. Baffioni, F. Beaudette, L. Benhabib, L. Bianchini, M. Bluj, P. Busson, C. Charlot, N. Daci, T. Dahms, M. Dalchenko, L. Dobrzynski, A. Florent, R. Granier de Cassagnac, M. Haguenauer, P. Miné, C. Mironov, I. N. Naranjo, M. Nguyen, C. Ochando, P. Paganini, D. Sabes, R. Salerno, Y. Sirois, C. Veelken, A. Zabi, J.-L. Agram, J. Andrea, D. Bloch, D. Bodin, J.-M. Brom, E. C. Chabert, C. Collard, E. Conte, F. Drouhin, J.-C. Fontaine, D. Gelé, U. Goerlach, C. Goetzmann, P. Juillot, A.-C. Le Bihan, P. Van Hove, S. Beauceron, N. Beaupere, O. Bondu, G. Boudoul, S. Brochet, J. Chasserat, R. Chierici, D. Contardo, P. Depasse, H. El Mamouni, J. Fay, S. Gascon, M. Gouzevitch, B. Ille, T. Kurca, M. Lethuillier, L. Mirabito, S. Perries, L. Sgandurra, V. Sordini, Y. Tschudi, M. Vander Donckt, P. Verdier, S. Viret, Z. Tsamalaidze, C. Autermann, S. Beranek, B. Calpas, M. Edelhoff, L. Feld, N. Heracleous, O. Hindrichs, R. Jussen, K. Klein, J. Merz, A. Ostapchuk, A. Perieanu, F. Raupach, J. Sammet, S. Schael, D. Sprenger, H. Weber, B. Wittmer, V. Zhukov, M. Ata, J. Caudron, E. Dietz-Laursonn, D. Duchardt, M. Erdmann, R. Fischer, A. Güth, T. Hebbeker, C. Heidemann, K. Hoepfner, D. Klingebiel, P. Kreuzer, M. Merschmeyer, A. Meyer, M. Olschewski, K. Padeken, P. Papacz, H. Pieta, H. Reithler, S. A. Schmitz, L. Sonnenschein, J. Steggemann, D. Teyssier, S. Thüer, M. Weber, M. Bontenackels, V. Cherepanov, Y. Erdogan, G. Flügge, H. Geenen, M. Geisler, W. Haj Ahmad, F. Hoehle, B. Kargoll, T. Kress, Y. Kuessel, J. Lingemann, A. Nowack, I. M. Nugent, L. Perchalla, O. Pooth, A. Stahl, M. Aldaya Martin, I. Asin, N. Bartosik, J. Behr, W. Behrenhoff, U. Behrens, M. Bergholz, A. Bethani, K. Borras, A. Burgmeier, A. Cakir, L. Calligaris, A. Campbell, F. Costanza, D. Dammann, C. Diez Pardos, T. Dorland, G. Eckerlin, D. Eckstein, G. Flucke, A. Geiser, I. Glushkov, P. Gunnellini, S. Habib, J. Hauk, G. Hellwig, H. Jung, M. Kasemann, P. Katsas, C. Kleinwort, H. Kluge, M. Krämer, D. Krücker, E. Kuznetsova, W. Lange, J. Leonard, W. Lohmann, B. Lutz, R. Mankel, I. Marfin, M. Marienfeld, I.-A. Melzer-Pellmann, A. B. Meyer, J. Mnich, A. Mussgiller, S. Naumann-Emme, O. Novgorodova, F. Nowak, J. Olzem, H. Perrey, A. Petrukhin, D. Pitzl, A. Raspereza, P. M. Ribeiro Cipriano, C. Riedl, E. Ron, M. Rosin, J. Salfeld-Nebgen, R. Schmidt, T. Schoerner-Sadenius, N. Sen, M. Stein, R. Walsh, C. Wissing, V. Blobel, H. Enderle, J. Erfle, U. Gebbert, M. Görner, M. Gosselink, J. Haller, R. S. Höing, K. Kaschube, G. Kaussen, H. Kirschenmann, R. Klanner, J. Lange, T. Peiffer, N. Pietsch, D. Rathjens, C. Sander, H. Schettler, P. Schleper, E. Schlieckau, A. Schmidt, T. Schum, M. Seidel, J. Sibille, V. Sola, H. Stadie, G. Steinbrück, J. Thomsen, L. Vanelderen, C. Barth, C. Baus, J. Berger, C. Böser, T. Chwalek, W. De Boer, A. Descroix, A. Dierlamm, M. Feindt, M. Guthoff, C. Hackstein, F. Hartmann, T. Hauth, M. Heinrich, H. Held, K. H. Hoffmann, U. Husemann, I. Katkov, J. R. Komaragiri, A. Kornmayer, P. Lobelle Pardo, D. Martschei, S. Mueller, Th. Müller, M. Niegel, A. Nürnberg, O. Oberst, J. Ott, G. Quast, K. Rabbertz, F. Ratnikov, N. Ratnikova, S. Röcker, F.-P. Schilling, G. Schott, H. J. Simonis, F. M. Stober, D. Troendle, R. Ulrich, J. Wagner-Kuhr, S. Wayand, T. Weiler, M. Zeise, G. Anagnostou, G. Daskalakis, T. Geralis, S. Kesisoglou, A. Kyriakis, D. Loukas, A. Markou, C. Markou, E. Ntomari, L. Gouskos, T. J. Mertzimekis, A. Panagiotou, N. Saoulidou, E. Stiliaris, X. Aslanoglou, I. Evangelou, G. Flouris, C. Foudas, P. Kokkas, N. Manthos, I. Papadopoulos, E. Paradas, G. Bencze, C. Hajdu, P. Hidas, D. Horvath, B. Radics, F. Sikler, V. Veszpremi, G. Vesztergombi, A. J. Zsigmond, N. Beni, S. Czellar, J. Molnar, J. Palinkas, Z. Szillasi, J. Karancsi, P. Raics, Z. L. Trocsanyi, B. Ujvari, S. B. Beri, V. Bhatnagar, N. Dhingra, R. Gupta, M. Kaur, M. Z. Mehta, M. Mittal, N. Nishu, L. K. Saini, A. Sharma, J. B. Singh, Ashok Kumar, Arun Kumar, S. Ahuja, A. Bhardwaj, B. C. Choudhary, S. Malhotra, M. Naimuddin, K. Ranjan, P. Saxena, V. Sharma, R. K. Shivpuri, S. Banerjee, S. Bhattacharya, K. Chatterjee, S. Dutta, B. Gomber, Sa. Jain, Sh. Jain, R. Khurana, A. Modak, S. Mukherjee, D. Roy, S. Sarkar, M. Sharan, A. Abdulsalam, D. Dutta, S. Kailas, V. Kumar, A. K. Mohanty, L. M. Pant, P. Shukla, A. Topkar, T. Aziz, R. M. Chatterjee, S. Ganguly, M. Guchait, A. Gurtu, M. Maity, G. Majumder, K. Mazumdar, G. B. Mohanty, B. Parida, K. Sudhakar, N. Wickramage, S. Banerjee, S. Dugad, H. Arfaei, H. Bakhshiansohi, S. M. Etesami, A. Fahim, H. Hesari, A. Jafari, M. Khakzad, M. Mohammadi Najafabadi, S. Paktinat Mehdiabadi, B. Safarzadeh, M. Zeinali, M. Abbrescia, L. Barbone, C. Calabria, S. S. Chhibra, A. Colaleo, D. Creanza, N. De Filippis, M. De Palma, L. Fiore, G. Iaselli, G. Maggi, M. Maggi, B. Marangelli, S. My, S. Nuzzo, N. Pacifico, A. Pompili, G. Pugliese, G. Selvaggi, L. Silvestris, G. Singh, R. Venditti, P. Verwilligen, G. Zito, G. Abbiendi, A. C. Benvenuti, D. Bonacorsi, S. Braibant-Giacomelli, L. Brigliadori, R. Campanini, P. Capiluppi, A. Castro, F. R. Cavallo, M. Cuffiani, G. M. Dallavalle, F. Fabbri, A. Fanfani, D. Fasanella, P. Giacomelli, C. Grandi, L. Guiducci, S. Marcellini, G. Masetti, M. Meneghelli, A. Montanari, F. L. Navarria, F. Odorici, A. Perrotta, F. Primavera, A. M. Rossi, T. Rovelli, G. P. Siroli, N. Tosi, R. Travaglini, S. Albergo, M. Chiorboli, S. Costa, R. Potenza, A. Tricomi, C. Tuve, G. Barbagli, V. Ciulli, C. Civinini, R. D’Alessandro, E. Focardi, S. Frosali, E. Gallo, S. Gonzi, P. Lenzi, M. Meschini, S. Paoletti, G. Sguazzoni, A. Tropiano, L. Benussi, S. Bianco, S. Colafranceschi, F. Fabbri, D. Piccolo, P. Fabbricatore, R. Musenich, S. Tosi, A. Benaglia, F. De Guio, L. Di Matteo, S. Fiorendi, S. Gennai, A. Ghezzi, M. T. Lucchini, S. Malvezzi, R. A. Manzoni, A. Martelli, A. Massironi, D. Menasce, L. Moroni, M. Paganoni, D. Pedrini, S. Ragazzi, N. Redaelli, T. Tabarelli de Fatis, S. Buontempo, N. Cavallo, A. De Cosa, O. Dogangun, F. Fabozzi, A. O. M. Iorio, L. Lista, S. Meola, M. Merola, P. Paolucci, P. Azzi, N. Bacchetta, P. Bellan, D. Bisello, A. Branca, R. Carlin, P. Checchia, T. Dorigo, U. Dosselli, M. Galanti, F. Gasparini, U. Gasparini, P. Giubilato, A. Gozzelino, K. Kanishchev, S. Lacaprara, I. Lazzizzera, M. Margoni, G. Maron, A. T. Meneguzzo, M. Nespolo, J. Pazzini, N. Pozzobon, P. Ronchese, F. Simonetto, E. Torassa, M. Tosi, S. Ventura, P. Zotto, G. Zumerle, M. Gabusi, S. P. Ratti, C. Riccardi, P. Vitulo, M. Biasini, G. M. Bilei, L. Fanò, P. Lariccia, G. Mantovani, M. Menichelli, A. Nappi, F. Romeo, A. Saha, A. Santocchia, A. Spiezia, S. Taroni, P. Azzurri, G. Bagliesi, T. Boccali, G. Broccolo, R. Castaldi, R. T. D’Agnolo, R. Dell’Orso, F. Fiori, L. Foà, A. Giassi, A. Kraan, F. Ligabue, T. Lomtadze, L. Martini, A. Messineo, F. Palla, A. Rizzi, A. T. Serban, P. Spagnolo, P. Squillacioti, R. Tenchini, G. Tonelli, A. Venturi, P. G. Verdini, C. Vernieri, L. Barone, F. Cavallari, D. Del Re, M. Diemoz, C. Fanelli, M. Grassi, E. Longo, F. Margaroli, P. Meridiani, F. Micheli, S. Nourbakhsh, G. Organtini, R. Paramatti, S. Rahatlou, L. Soffi, N. Amapane, R. Arcidiacono, S. Argiro, M. Arneodo, C. Biino, N. Cartiglia, S. Casasso, M. Costa, N. Demaria, C. Mariotti, S. Maselli, E. Migliore, V. Monaco, M. Musich, M. M. Obertino, N. Pastrone, M. Pelliccioni, A. Potenza, A. Romero, M. Ruspa, R. Sacchi, A. Solano, A. Staiano, U. Tamponi, S. Belforte, V. Candelise, M. Casarsa, F. Cossutti, G. Della Ricca, B. Gobbo, M. Marone, D. Montanino, A. Penzo, A. Schizzi, A. Zanetti, T. Y. Kim, S. K. Nam, S. Chang, D. H. Kim, G. N. Kim, J. E. Kim, D. J. Kong, Y. D. Oh, H. Park, D. C. Son, J. Y. Kim, Zero J. Kim, S. Song, S. Choi, D. Gyun, B. Hong, M. Jo, H. Kim, T. J. Kim, K. S. Lee, D. H. Moon, S. K. Park, Y. Roh, M. Choi, J. H. Kim, C. Park, I. C. Park, S. Park, G. Ryu, Y. Choi, Y. K. Choi, J. Goh, M. S. Kim, E. Kwon, B. Lee, J. Lee, S. Lee, H. Seo, I. Yu, I. Grigelionis, A. Juodagalvis, H. Castilla-Valdez, E. De La Cruz-Burelo, I. Heredia-de La Cruz, R. Lopez-Fernandez, J. Martínez-Ortega, A. Sanchez-Hernandez, L. M. Villasenor-Cendejas, S. Carrillo Moreno, F. Vazquez Valencia, H. A. Salazar Ibarguen, E. Casimiro Linares, A. Morelos Pineda, M. A. Reyes-Santos, D. Krofcheck, A. J. Bell, P. H. Butler, R. Doesburg, S. Reucroft, H. Silverwood, M. Ahmad, M. I. Asghar, J. Butt, H. R. Hoorani, S. Khalid, W. A. Khan, T. Khurshid, S. Qazi, M. A. Shah, M. Shoaib, H. Bialkowska, B. Boimska, T. Frueboes, M. Górski, M. Kazana, K. Nawrocki, K. Romanowska-Rybinska, M. Szleper, G. Wrochna, P. Zalewski, G. Brona, K. Bunkowski, M. Cwiok, W. Dominik, K. Doroba, A. Kalinowski, M. Konecki, J. Krolikowski, M. Misiura, W. Wolszczak, N. Almeida, P. Bargassa, A. David, P. Faccioli, P. G. Ferreira Parracho, M. Gallinaro, J. Seixas, J. Varela, P. Vischia, P. Bunin, I. Golutvin, I. Gorbunov, V. Karjavin, V. Konoplyanikov, G. Kozlov, A. Lanev, A. Malakhov, P. Moisenz, V. Palichik, V. Perelygin, M. Savina, S. Shmatov, S. Shulha, N. Skatchkov, V. Smirnov, A. Zarubin, S. Evstyukhin, V. Golovtsov, Y. Ivanov, V. Kim, P. Levchenko, V. Murzin, V. Oreshkin, I. Smirnov, V. Sulimov, L. Uvarov, S. Vavilov, A. Vorobyev, An. Vorobyev, Yu. Andreev, A. Dermenev, S. Gninenko, N. Golubev, M. Kirsanov, N. Krasnikov, V. Matveev, A. Pashenkov, D. Tlisov, A. Toropin, V. Epshteyn, M. Erofeeva, V. Gavrilov, N. Lychkovskaya, V. Popov, G. Safronov, S. Semenov, A. Spiridonov, V. Stolin, E. Vlasov, A. Zhokin, V. Andreev, M. Azarkin, I. Dremin, M. Kirakosyan, A. Leonidov, G. Mesyats, S. V. Rusakov, A. Vinogradov, A. Belyaev, E. Boos, V. Bunichev, M. Dubinin, L. Dudko, A. Ershov, A. Gribushin, V. Klyukhin, O. Kodolova, I. Lokhtin, A. Markina, S. Obraztsov, V. Savrin, A. Snigirev, I. Azhgirey, I. Bayshev, S. Bitioukov, V. Kachanov, A. Kalinin, D. Konstantinov, V. Krychkine, V. Petrov, R. Ryutin, A. Sobol, L. Tourtchanovitch, S. Troshin, N. Tyurin, A. Uzunian, A. Volkov, P. Adzic, M. Ekmedzic, D. Krpic, J. Milosevic, M. Aguilar-Benitez, J. Alcaraz Maestre, C. Battilana, E. Calvo, M. Cerrada, M. Chamizo Llatas, N. Colino, B. De La Cruz, A. Delgado Peris, D. Domínguez Vázquez, C. Fernandez Bedoya, J. P. Fernández Ramos, A. Ferrando, J. Flix, M. C. Fouz, P. Garcia-Abia, O. Gonzalez Lopez, S. Goy Lopez, J. M. Hernandez, M. I. Josa, G. Merino, J. Puerta Pelayo, A. Quintario Olmeda, I. Redondo, L. Romero, J. Santaolalla, M. S. Soares, C. Willmott, C. Albajar, J. F. de Trocóniz, H. Brun, J. Cuevas, J. Fernandez Menendez, S. Folgueras, I. Gonzalez Caballero, L. Lloret Iglesias, J. Piedra Gomez, J. A. Brochero Cifuentes, I. J. Cabrillo, A. Calderon, S. H. Chuang, J. Duarte Campderros, M. Fernandez, G. Gomez, J. Gonzalez Sanchez, A. Graziano, C. Jorda, A. Lopez Virto, J. Marco, R. Marco, C. Martinez Rivero, F. Matorras, F. J. Munoz Sanchez, T. Rodrigo, A. Y. Rodríguez-Marrero, A. Ruiz-Jimeno, L. Scodellaro, I. Vila, R. Vilar Cortabitarte, D. Abbaneo, E. Auffray, G. Auzinger, M. Bachtis, P. Baillon, A. H. Ball, D. Barney, J. Bendavid, J. F. Benitez, C. Bernet, G. Bianchi, P. Bloch, A. Bocci, A. Bonato, C. Botta, H. Breuker, T. Camporesi, G. Cerminara, T. Christiansen, J. A. Coarasa Perez, D. d’Enterria, A. Dabrowski, A. De Roeck, S. De Visscher, S. Di Guida, M. Dobson, N. Dupont-Sagorin, A. Elliott-Peisert, J. Eugster, W. Funk, G. Georgiou, M. Giffels, D. Gigi, K. Gill, D. Giordano, M. Giunta, F. Glege, R. Gomez-Reino Garrido, P. Govoni, S. Gowdy, R. Guida, J. Hammer, M. Hansen, P. Harris, C. Hartl, J. Harvey, B. Hegner, A. Hinzmann, V. Innocente, P. Janot, K. Kaadze, E. Karavakis, K. Kousouris, K. Krajczar, P. Lecoq, Y.-J. Lee, C. Lourenço, M. Malberti, L. Malgeri, M. Mannelli, L. Masetti, F. Meijers, S. Mersi, E. Meschi, R. Moser, M. Mulders, P. Musella, E. Nesvold, L. Orsini, E. Palencia Cortezon, E. Perez, L. Perrozzi, A. Petrilli, A. Pfeiffer, M. Pierini, M. Pimiä, D. Piparo, G. Polese, L. Quertenmont, A. Racz, W. Reece, J. Rodrigues Antunes, G. Rolandi, C. Rovelli, M. Rovere, H. Sakulin, F. Santanastasio, C. Schäfer, C. Schwick, I. Segoni, S. Sekmen, A. Sharma, P. Siegrist, P. Silva, M. Simon, P. Sphicas, D. Spiga, M. Stoye, A. Tsirou, G. I. Veres, J. R. Vlimant, H. K. Wöhri, S. D. Worm, W. D. Zeuner, W. Bertl, K. Deiters, W. Erdmann, K. Gabathuler, R. Horisberger, Q. Ingram, H. C. Kaestli, S. König, D. Kotlinski, U. Langenegger, F. Meier, D. Renker, T. Rohe, F. Bachmair, L. Bäni, P. Bortignon, M. A. Buchmann, B. Casal, N. Chanon, A. Deisher, G. Dissertori, M. Dittmar, M. Donegà, M. Dünser, P. Eller, C. Grab, D. Hits, P. Lecomte, W. Lustermann, A. C. Marini, P. Martinez Ruiz del Arbol, N. Mohr, F. Moortgat, C. Nägeli, P. Nef, F. Nessi-Tedaldi, F. Pandolfi, L. Pape, F. Pauss, M. Peruzzi, F. J. Ronga, M. Rossini, L. Sala, A. K. Sanchez, A. Starodumov, B. Stieger, M. Takahashi, L. Tauscher, A. Thea, K. Theofilatos, D. Treille, C. Urscheler, R. Wallny, H. A. Weber, C. Amsler, V. Chiochia, C. Favaro, M. Ivova Rikova, B. Kilminster, B. Millan Mejias, P. Otiougova, P. Robmann, H. Snoek, S. Tupputi, M. Verzetti, M. Cardaci, K. H. Chen, C. Ferro, C. M. Kuo, S. W. Li, W. Lin, Y. J. Lu, R. Volpe, S. S. Yu, P. Bartalini, P. Chang, Y. H. Chang, Y. W. Chang, Y. Chao, K. F. Chen, C. Dietz, U. Grundler, W.-S. Hou, Y. Hsiung, K. Y. Kao, Y. J. Lei, R.-S. Lu, D. Majumder, E. Petrakou, X. Shi, J. G. Shiu, Y. M. Tzeng, M. Wang, B. Asavapibhop, N. Suwonjandee, A. Adiguzel, M. N. Bakirci, S. Cerci, C. Dozen, I. Dumanoglu, E. Eskut, S. Girgis, G. Gokbulut, E. Gurpinar, I. Hos, E. E. Kangal, A. Kayis Topaksu, G. Onengut, K. Ozdemir, S. Ozturk, A. Polatoz, K. Sogut, D. Sunar Cerci, B. Tali, H. Topakli, M. Vergili, I. V. Akin, T. Aliev, B. Bilin, S. Bilmis, M. Deniz, H. Gamsizkan, A. M. Guler, G. Karapinar, K. Ocalan, A. Ozpineci, M. Serin, R. Sever, U. E. Surat, M. Yalvac, M. Zeyrek, E. Gülmez, B. Isildak, M. Kaya, O. Kaya, S. Ozkorucuklu, N. Sonmez, H. Bahtiyar, E. Barlas, K. Cankocak, Y. O. Günaydin, F. I. Vardarlı, M. Yücel, L. Levchuk, P. Sorokin, J. J. Brooke, E. Clement, D. Cussans, H. Flacher, R. Frazier, J. Goldstein, M. Grimes, G. P. Heath, H. F. Heath, L. Kreczko, C. Lucas, Z. Meng, S. Metson, D. M. Newbold, K. Nirunpong, A. Poll, S. Senkin, V. J. Smith, T. Williams, L. Basso, K. W. Bell, A. Belyaev, C. Brew, R. M. Brown, D. J. A. Cockerill, J. A. Coughlan, K. Harder, S. Harper, J. Jackson, E. Olaiya, D. Petyt, B. C. Radburn-Smith, C. H. Shepherd-Themistocleous, I. R. Tomalin, W. J. Womersley, R. Bainbridge, G. Ball, O. Buchmuller, D. Burton, D. Colling, N. Cripps, M. Cutajar, P. Dauncey, G. Davies, M. Della Negra, W. Ferguson, J. Fulcher, A. Gilbert, A. Guneratne Bryer, G. Hall, Z. Hatherell, J. Hays, G. Iles, M. Jarvis, G. Karapostoli, M. Kenzie, L. Lyons, A.-M. Magnan, J. Marrouche, B. Mathias, R. Nandi, J. Nash, A. Nikitenko, J. Pela, M. Pesaresi, K. Petridis, M. Pioppi, D. M. Raymond, S. Rogerson, A. Rose, C. Seez, P. Sharp, A. Sparrow, A. Tapper, M. Vazquez Acosta, T. Virdee, S. Wakefield, N. Wardle, T. Whyntie, M. Chadwick, J. E. Cole, P. R. Hobson, A. Khan, P. Kyberd, D. Leggat, D. Leslie, W. Martin, I. D. Reid, P. Symonds, L. Teodorescu, M. Turner, J. Dittmann, K. Hatakeyama, A. Kasmi, H. Liu, T. Scarborough, O. Charaf, S. I. Cooper, C. Henderson, P. Rumerio, A. Avetisyan, T. Bose, C. Fantasia, A. Heister, P. Lawson, D. Lazic, J. Rohlf, D. Sperka, J. St. John, L. Sulak, J. Alimena, S. Bhattacharya, G. Christopher, D. Cutts, Z. Demiragli, A. Ferapontov, A. Garabedian, U. Heintz, G. Kukartsev, E. Laird, G. Landsberg, M. Luk, M. Narain, M. Segala, T. Sinthuprasith, T. Speer, R. Breedon, G. Breto, M. Calderon De La Barca Sanchez, M. Caulfield, S. Chauhan, M. Chertok, J. Conway, R. Conway, P. T. Cox, J. Dolen, R. Erbacher, M. Gardner, R. Houtz, W. Ko, A. Kopecky, R. Lander, O. Mall, T. Miceli, R. Nelson, D. Pellett, F. Ricci-Tam, B. Rutherford, M. Searle, J. Smith, M. Squires, M. Tripathi, R. Yohay, V. Andreev, D. Cline, R. Cousins, J. Duris, S. Erhan, P. Everaerts, C. Farrell, M. Felcini, J. Hauser, M. Ignatenko, C. Jarvis, G. Rakness, P. Schlein, P. Traczyk, V. Valuev, M. Weber, J. Babb, R. Clare, M. E. Dinardo, J. Ellison, J. W. Gary, F. Giordano, G. Hanson, H. Liu, O. R. Long, A. Luthra, H. Nguyen, S. Paramesvaran, J. Sturdy, S. Sumowidagdo, R. Wilken, S. Wimpenny, W. Andrews, J. G. Branson, G. B. Cerati, S. Cittolin, D. Evans, A. Holzner, R. Kelley, M. Lebourgeois, J. Letts, I. Macneill, B. Mangano, S. Padhi, C. Palmer, G. Petrucciani, M. Pieri, M. Sani, V. Sharma, S. Simon, E. Sudano, M. Tadel, Y. Tu, A. Vartak, S. Wasserbaech, F. Würthwein, A. Yagil, J. Yoo, D. Barge, R. Bellan, C. Campagnari, M. D’Alfonso, T. Danielson, K. Flowers, P. Geffert, C. George, F. Golf, J. Incandela, C. Justus, P. Kalavase, D. Kovalskyi, V. Krutelyov, S. Lowette, R. Magaña Villalba, N. Mccoll, V. Pavlunin, J. Ribnik, J. Richman, R. Rossin, D. Stuart, W. To, C. West, A. Apresyan, A. Bornheim, J. Bunn, Y. Chen, E. Di Marco, J. Duarte, D. Kcira, Y. Ma, A. Mott, H. B. Newman, C. Rogan, M. Spiropulu, V. Timciuc, J. Veverka, R. Wilkinson, S. Xie, Y. Yang, R. Y. Zhu, V. Azzolini, A. Calamba, R. Carroll, T. Ferguson, Y. Iiyama, D. W. Jang, Y. F. Liu, M. Paulini, J. Russ, H. Vogel, I. Vorobiev, J. P. Cumalat, B. R. Drell, W. T. Ford, A. Gaz, E. Luiggi Lopez, U. Nauenberg, J. G. Smith, K. Stenson, K. A. Ulmer, S. R. Wagner, J. Alexander, A. Chatterjee, N. Eggert, L. K. Gibbons, W. Hopkins, A. Khukhunaishvili, B. Kreis, N. Mirman, G. Nicolas Kaufman, J. R. Patterson, A. Ryd, E. Salvati, W. Sun, W. D. Teo, J. Thom, J. Thompson, J. Tucker, Y. Weng, L. Winstrom, P. Wittich, D. Winn, S. Abdullin, M. Albrow, J. Anderson, G. Apollinari, L. A. T. Bauerdick, A. Beretvas, J. Berryhill, P. C. Bhat, K. Burkett, J. N. Butler, V. Chetluru, H. W. K. Cheung, F. Chlebana, S. Cihangir, V. D. Elvira, I. Fisk, J. Freeman, Y. Gao, E. Gottschalk, L. Gray, D. Green, O. Gutsche, R. M. Harris, J. Hirschauer, B. Hooberman, S. Jindariani, M. Johnson, U. Joshi, B. Klima, S. Kunori, S. Kwan, J. Linacre, D. Lincoln, R. Lipton, J. Lykken, K. Maeshima, J. M. Marraffino, V. I. Martinez Outschoorn, S. Maruyama, D. Mason, P. McBride, K. Mishra, S. Mrenna, Y. Musienko, C. Newman-Holmes, V. O’Dell, O. Prokofyev, E. Sexton-Kennedy, S. Sharma, W. J. Spalding, L. Spiegel, L. Taylor, S. Tkaczyk, N. V. Tran, L. Uplegger, E. W. Vaandering, R. Vidal, J. Whitmore, W. Wu, F. Yang, J. C. Yun, D. Acosta, P. Avery, D. Bourilkov, M. Chen, T. Cheng, S. Das, M. De Gruttola, G. P. Di Giovanni, D. Dobur, A. Drozdetskiy, R. D. Field, M. Fisher, Y. Fu, I. K. Furic, J. Hugon, B. Kim, J. Konigsberg, A. Korytov, A. Kropivnitskaya, T. Kypreos, J. F. Low, K. Matchev, P. Milenovic, G. Mitselmakher, L. Muniz, R. Remington, A. Rinkevicius, N. Skhirtladze, M. Snowball, J. Yelton, M. Zakaria, V. Gaultney, S. Hewamanage, L. M. Lebolo, S. Linn, P. Markowitz, G. Martinez, J. L. Rodriguez, T. Adams, A. Askew, J. Bochenek, J. Chen, B. Diamond, S. V. Gleyzer, J. Haas, S. Hagopian, V. Hagopian, K. F. Johnson, H. Prosper, V. Veeraraghavan, M. Weinberg, M. M. Baarmand, B. Dorney, M. Hohlmann, H. Kalakhety, F. Yumiceva, M. R. Adams, L. Apanasevich, V. E. Bazterra, R. R. Betts, I. Bucinskaite, J. Callner, R. Cavanaugh, O. Evdokimov, L. Gauthier, C. E. Gerber, D. J. Hofman, S. Khalatyan, P. Kurt, F. Lacroix, C. O’Brien, C. Silkworth, D. Strom, P. Turner, N. Varelas, U. Akgun, E. A. Albayrak, B. Bilki, W. Clarida, K. Dilsiz, F. Duru, S. Griffiths, J.-P. Merlo, H. Mermerkaya, A. Mestvirishvili, A. Moeller, J. Nachtman, C. R. Newsom, H. Ogul, Y. Onel, F. Ozok, S. Sen, P. Tan, E. Tiras, J. Wetzel, T. Yetkin, K. Yi, B. A. Barnett, B. Blumenfeld, S. Bolognesi, D. Fehling, G. Giurgiu, A. V. Gritsan, G. Hu, P. Maksimovic, M. Swartz, A. Whitbeck, P. Baringer, A. Bean, G. Benelli, R. P. Kenny Iii, M. Murray, D. Noonan, S. Sanders, R. Stringer, J. S. Wood, A. F. Barfuss, I. Chakaberia, A. Ivanov, S. Khalil, M. Makouski, Y. Maravin, S. Shrestha, I. Svintradze, J. Gronberg, D. Lange, F. Rebassoo, D. Wright, A. Baden, B. Calvert, S. C. Eno, J. A. Gomez, N. J. Hadley, R. G. Kellogg, T. Kolberg, Y. Lu, M. Marionneau, A. C. Mignerey, K. Pedro, A. Peterman, A. Skuja, J. Temple, M. B. Tonjes, S. C. Tonwar, A. Apyan, G. Bauer, W. Busza, E. Butz, I. A. Cali, M. Chan, V. Dutta, G. Gomez Ceballos, M. Goncharov, Y. Kim, M. Klute, A. Levin, P. D. Luckey, T. Ma, S. Nahn, C. Paus, D. Ralph, C. Roland, G. Roland, G. S. F. Stephans, F. Stöckli, K. Sumorok, K. Sung, D. Velicanu, R. Wolf, B. Wyslouch, M. Yang, Y. Yilmaz, A. S. Yoon, M. Zanetti, V. Zhukova, B. Dahmes, A. De Benedetti, G. Franzoni, A. Gude, J. Haupt, S. C. Kao, K. Klapoetke, Y. Kubota, J. Mans, N. Pastika, R. Rusack, M. Sasseville, A. Singovsky, N. Tambe, J. Turkewitz, L. M. Cremaldi, R. Kroeger, L. Perera, R. Rahmat, D. A. Sanders, D. Summers, E. Avdeeva, K. Bloom, S. Bose, D. R. Claes, A. Dominguez, M. Eads, J. Keller, I. Kravchenko, J. Lazo-Flores, S. Malik, G. R. Snow, A. Godshalk, I. Iashvili, S. Jain, A. Kharchilava, A. Kumar, S. Rappoccio, Z. Wan, G. Alverson, E. Barberis, D. Baumgartel, M. Chasco, J. Haley, D. Nash, T. Orimoto, D. Trocino, D. Wood, J. Zhang, A. Anastassov, K. A. Hahn, A. Kubik, L. Lusito, N. Mucia, N. Odell, B. Pollack, A. Pozdnyakov, M. Schmitt, S. Stoynev, M. Velasco, S. Won, D. Berry, A. Brinkerhoff, K. M. Chan, M. Hildreth, C. Jessop, D. J. Karmgard, J. Kolb, K. Lannon, W. Luo, S. Lynch, N. Marinelli, D. M. Morse, T. Pearson, M. Planer, R. Ruchti, J. Slaunwhite, N. Valls, M. Wayne, M. Wolf, L. Antonelli, B. Bylsma, L. S. Durkin, C. Hill, R. Hughes, K. Kotov, T. Y. Ling, D. Puigh, M. Rodenburg, G. Smith, C. Vuosalo, G. Williams, B. L. Winer, H. Wolfe, E. Berry, P. Elmer, V. Halyo, P. Hebda, J. Hegeman, A. Hunt, P. Jindal, S. A. Koay, D. Lopes Pegna, P. Lujan, D. Marlow, T. Medvedeva, M. Mooney, J. Olsen, P. Piroué, X. Quan, A. Raval, H. Saka, D. Stickland, C. Tully, J. S. Werner, S. C. Zenz, A. Zuranski, E. Brownson, A. Lopez, H. Mendez, J. E. Ramirez Vargas, E. Alagoz, D. Benedetti, G. Bolla, D. Bortoletto, M. De Mattia, A. Everett, Z. Hu, M. Jones, O. Koybasi, M. Kress, N. Leonardo, V. Maroussov, P. Merkel, D. H. Miller, N. Neumeister, I. Shipsey, D. Silvers, A. Svyatkovskiy, M. Vidal Marono, H. D. Yoo, J. Zablocki, Y. Zheng, S. Guragain, N. Parashar, A. Adair, B. Akgun, K. M. Ecklund, F. J. M. Geurts, W. Li, B. P. Padley, R. Redjimi, J. Roberts, J. Zabel, B. Betchart, A. Bodek, R. Covarelli, P. de Barbaro, R. Demina, Y. Eshaq, T. Ferbel, A. Garcia-Bellido, P. Goldenzweig, J. Han, A. Harel, D. C. Miner, G. Petrillo, D. Vishnevskiy, M. Zielinski, A. Bhatti, R. Ciesielski, L. Demortier, K. Goulianos, G. Lungu, S. Malik, C. Mesropian, S. Arora, A. Barker, J. P. Chou, C. Contreras-Campana, E. Contreras-Campana, D. Duggan, D. Ferencek, Y. Gershtein, R. Gray, E. Halkiadakis, D. Hidas, A. Lath, S. Panwalkar, M. Park, R. Patel, V. Rekovic, J. Robles, K. Rose, S. Salur, S. Schnetzer, C. Seitz, S. Somalwar, R. Stone, M. Walker, G. Cerizza, M. Hollingsworth, S. Spanier, Z. C. Yang, A. York, R. Eusebi, W. Flanagan, J. Gilmore, T. Kamon, V. Khotilovich, R. Montalvo, I. Osipenkov, Y. Pakhotin, A. Perloff, J. Roe, A. Safonov, T. Sakuma, I. Suarez, A. Tatarinov, D. Toback, N. Akchurin, J. Damgov, C. Dragoiu, P. R. Dudero, C. Jeong, K. Kovitanggoon, S. W. Lee, T. Libeiro, I. Volobouev, E. Appelt, A. G. Delannoy, S. Greene, A. Gurrola, W. Johns, C. Maguire, Y. Mao, A. Melo, M. Sharma, P. Sheldon, B. Snook, S. Tuo, J. Velkovska, M. W. Arenton, M. Balazs, S. Boutle, B. Cox, B. Francis, J. Goodell, R. Hirosky, A. Ledovskoy, C. Lin, C. Neu, J. Wood, S. Gollapinni, R. Harr, P. E. Karchin, C. Kottachchi Kankanamge Don, P. Lamichhane, A. Sakharov, M. Anderson, D. A. Belknap, L. Borrello, D. Carlsmith, M. Cepeda, S. Dasu, E. Friis, K. S. Grogg, M. Grothe, R. Hall-Wilton, M. Herndon, A. Hervé, P. Klabbers, J. Klukas, A. Lanaro, C. Lazaridis, R. Loveless, A. Mohapatra, M. U. Mozer, I. Ojalvo, G. A. Pierro, I. Ross, A. Savin, W. H. Smith, J. Swanson

**Affiliations:** 1 CERN, Geneva, Switzerland; 2 Yerevan Physics Institute, Yerevan, Armenia; 3 Institut für Hochenergiephysik der OeAW, Wien, Austria; 4 National Centre for Particle and High Energy Physics, Minsk, Belarus; 5 Universiteit Antwerpen, Antwerpen, Belgium; 6 Vrije Universiteit Brussel, Brussel, Belgium; 7 Université Libre de Bruxelles, Bruxelles, Belgium; 8 Ghent University, Ghent, Belgium; 9 Université Catholique de Louvain, Louvain-la-Neuve, Belgium; 10 Université de Mons, Mons, Belgium; 11 Centro Brasileiro de Pesquisas Fisicas, Rio de Janeiro, Brazil; 12 Universidade do Estado do Rio de Janeiro, Rio de Janeiro, Brazil; 13 Universidade Estadual Paulista, São Paulo, Brazil; 14 Universidade Federal do ABC, São Paulo, Brazil; 15 Institute for Nuclear Research and Nuclear Energy, Sofia, Bulgaria; 16 University of Sofia, Sofia, Bulgaria; 17 Institute of High Energy Physics, Beijing, China; 18 State Key Laboratory of Nuclear Physics and Technology, Peking University, Beijing, China; 19 Universidad de Los Andes, Bogota, Colombia; 20 Technical University of Split, Split, Croatia; 21 University of Split, Split, Croatia; 22 Institute Rudjer Boskovic, Zagreb, Croatia; 23 University of Cyprus, Nicosia, Cyprus; 24 Charles University, Prague, Czech Republic; 25 Academy of Scientific Research and Technology of the Arab Republic of Egypt, Egyptian Network of High Energy Physics, Cairo, Egypt; 26 National Institute of Chemical Physics and Biophysics, Tallinn, Estonia; 27 Department of Physics, University of Helsinki, Helsinki, Finland; 28 Helsinki Institute of Physics, Helsinki, Finland; 29 Lappeenranta University of Technology, Lappeenranta, Finland; 30 DSM/IRFU, CEA/Saclay, Gif-sur-Yvette, France; 31 Laboratoire Leprince-Ringuet, Ecole Polytechnique, IN2P3-CNRS, Palaiseau, France; 32 Institut Pluridisciplinaire Hubert Curien, Université de Strasbourg, Université de Haute Alsace Mulhouse, CNRS/IN2P3, Strasbourg, France; 33 CNRS-IN2P3, Institut de Physique Nucléaire de Lyon, Université de Lyon, Université Claude Bernard Lyon 1, Villeurbanne, France; 34 Institute of High Energy Physics and Informatization, Tbilisi State University, Tbilisi, Georgia; 35 I. Physikalisches Institut, RWTH Aachen University, Aachen, Germany; 36 III. Physikalisches Institut A, RWTH Aachen University, Aachen, Germany; 37 III. Physikalisches Institut B, RWTH Aachen University, Aachen, Germany; 38 Deutsches Elektronen-Synchrotron, Hamburg, Germany; 39 University of Hamburg, Hamburg, Germany; 40 Institut für Experimentelle Kernphysik, Karlsruhe, Germany; 41 Institute of Nuclear and Particle Physics (INPP), NCSR Demokritos, Aghia Paraskevi, Greece; 42 University of Athens, Athens, Greece; 43 University of Ioánnina, Ioánnina, Greece; 44 KFKI Research Institute for Particle and Nuclear Physics, Budapest, Hungary; 45 Institute of Nuclear Research ATOMKI, Debrecen, Hungary; 46 University of Debrecen, Debrecen, Hungary; 47 Panjab University, Chandigarh, India; 48 University of Delhi, Delhi, India; 49 Saha Institute of Nuclear Physics, Kolkata, India; 50 Bhabha Atomic Research Centre, Mumbai, India; 51 Tata Institute of Fundamental Research - EHEP, Mumbai, India; 52 Tata Institute of Fundamental Research - HECR, Mumbai, India; 53 Institute for Research in Fundamental Sciences (IPM), Tehran, Iran; 54 INFN Sezione di Bari, Bari, Italy; 55 Università di Bari, Bari, Italy; 56 Politecnico di Bari, Bari, Italy; 57 INFN Sezione di Bologna, Bologna, Italy; 58 Università di Bologna, Bologna, Italy; 59 INFN Sezione di Catania, Catania, Italy; 60 Università di Catania, Catania, Italy; 61 INFN Sezione di Firenze, Firenze, Italy; 62 Università di Firenze, Firenze, Italy; 63 INFN Laboratori Nazionali di Frascati, Frascati, Italy; 64 INFN Sezione di Genova, Genova, Italy; 65 Università di Genova, Genova, Italy; 66 INFN Sezione di Milano-Bicocca, Milano, Italy; 67 Università di Milano-Bicocca, Milano, Italy; 68 INFN Sezione di Napoli, Napoli, Italy; 69 Università di Napoli ’Federico II’, Napoli, Italy; 70 Università della Basilicata (Potenza), Napoli, Italy; 71 Università G. Marconi (Roma), Napoli, Italy; 72 INFN Sezione di Padova, Padova, Italy; 73 Università di Padova, Padova, Italy; 74 Università di Trento (Trento), Padova, Italy; 75 INFN Sezione di Pavia, Pavia, Italy; 76 Università di Pavia, Pavia, Italy; 77 INFN Sezione di Perugia, Perugia, Italy; 78 Università di Perugia, Perugia, Italy; 79 INFN Sezione di Pisa, Pisa, Italy; 80 Università di Pisa, Pisa, Italy; 81 Scuola Normale Superiore di Pisa, Pisa, Italy; 82 INFN Sezione di Roma, Roma, Italy; 83 Università di Roma, Roma, Italy; 84 INFN Sezione di Torino, Torino, Italy; 85 Università di Torino, Torino, Italy; 86 Università del Piemonte Orientale (Novara), Torino, Italy; 87 INFN Sezione di Trieste, Trieste, Italy; 88 Università di Trieste, Trieste, Italy; 89 Kangwon National University, Chunchon, Korea; 90 Kyungpook National University, Daegu, Korea; 91 Institute for Universe and Elementary Particles, Chonnam National University, Kwangju, Korea; 92 Korea University, Seoul, Korea; 93 University of Seoul, Seoul, Korea; 94 Sungkyunkwan University, Suwon, Korea; 95 Vilnius University, Vilnius, Lithuania; 96 Centro de Investigacion y de Estudios Avanzados del IPN, Mexico City, Mexico; 97 Universidad Iberoamericana, Mexico City, Mexico; 98 Benemerita Universidad Autonoma de Puebla, Puebla, Mexico; 99 Universidad Autónoma de San Luis Potosí, San Luis Potosí, Mexico; 100 University of Auckland, Auckland, New Zealand; 101 University of Canterbury, Christchurch, New Zealand; 102 National Centre for Physics, Quaid-I-Azam University, Islamabad, Pakistan; 103 National Centre for Nuclear Research, Swierk, Poland; 104 Institute of Experimental Physics, Faculty of Physics, University of Warsaw, Warsaw, Poland; 105 Laboratório de Instrumentação e Física Experimental de Partículas, Lisboa, Portugal; 106 Joint Institute for Nuclear Research, Dubna, Russia; 107 Petersburg Nuclear Physics Institute, Gatchina (St. Petersburg), Russia; 108 Institute for Nuclear Research, Moscow, Russia; 109 Institute for Theoretical and Experimental Physics, Moscow, Russia; 110 P.N. Lebedev Physical Institute, Moscow, Russia; 111 Skobeltsyn Institute of Nuclear Physics, Lomonosov Moscow State University, Moscow, Russia; 112 State Research Center of Russian Federation, Institute for High Energy Physics, Protvino, Russia; 113 Faculty of Physics and Vinca Institute of Nuclear Sciences, University of Belgrade, Belgrade, Serbia; 114 Centro de Investigaciones Energéticas Medioambientales y Tecnológicas (CIEMAT), Madrid, Spain; 115 Universidad Autónoma de Madrid, Madrid, Spain; 116 Universidad de Oviedo, Oviedo, Spain; 117 Instituto de Física de Cantabria (IFCA), CSIC-Universidad de Cantabria, Santander, Spain; 118 European Organization for Nuclear Research, CERN, Geneva, Switzerland; 119 Paul Scherrer Institut, Villigen, Switzerland; 120 Institute for Particle Physics, ETH Zurich, Zurich, Switzerland; 121 Universität Zürich, Zurich, Switzerland; 122 National Central University, Chung-Li, Taiwan; 123 National Taiwan University (NTU), Taipei, Taiwan; 124 Chulalongkorn University, Bangkok, Thailand; 125 Cukurova University, Adana, Turkey; 126 Physics Department, Middle East Technical University, Ankara, Turkey; 127 Bogazici University, Istanbul, Turkey; 128 Istanbul Technical University, Istanbul, Turkey; 129 National Scientific Center, Kharkov Institute of Physics and Technology, Kharkov, Ukraine; 130 University of Bristol, Bristol, United Kingdom; 131 Rutherford Appleton Laboratory, Didcot, United Kingdom; 132 Imperial College, London, United Kingdom; 133 Brunel University, Uxbridge, United Kingdom; 134 Baylor University, Waco, USA; 135 The University of Alabama, Tuscaloosa, USA; 136 Boston University, Boston, USA; 137 Brown University, Providence, USA; 138 University of California, Davis, Davis, USA; 139 University of California, Los Angeles, USA; 140 University of California, Riverside, Riverside, USA; 141 University of California, San Diego, La Jolla, USA; 142 University of California, Santa Barbara, Santa Barbara, USA; 143 California Institute of Technology, Pasadena, USA; 144 Carnegie Mellon University, Pittsburgh, USA; 145 University of Colorado at Boulder, Boulder, USA; 146 Cornell University, Ithaca, USA; 147 Fairfield University, Fairfield, USA; 148 Fermi National Accelerator Laboratory, Batavia, USA; 149 University of Florida, Gainesville, USA; 150 Florida International University, Miami, USA; 151 Florida State University, Tallahassee, USA; 152 Florida Institute of Technology, Melbourne, USA; 153 University of Illinois at Chicago (UIC), Chicago, USA; 154 The University of Iowa, Iowa City, USA; 155 Johns Hopkins University, Baltimore, USA; 156 The University of Kansas, Lawrence, USA; 157 Kansas State University, Manhattan, USA; 158 Lawrence Livermore National Laboratory, Livermore, USA; 159 University of Maryland, College Park, USA; 160 Massachusetts Institute of Technology, Cambridge, USA; 161 University of Minnesota, Minneapolis, USA; 162 University of Mississippi, Oxford, USA; 163 University of Nebraska-Lincoln, Lincoln, USA; 164 State University of New York at Buffalo, Buffalo, USA; 165 Northeastern University, Boston, USA; 166 Northwestern University, Evanston, USA; 167 University of Notre Dame, Notre Dame, USA; 168 The Ohio State University, Columbus, USA; 169 Princeton University, Princeton, USA; 170 University of Puerto Rico, Mayaguez, USA; 171 Purdue University, West Lafayette, USA; 172 Purdue University Calumet, Hammond, USA; 173 Rice University, Houston, USA; 174 University of Rochester, Rochester, USA; 175 The Rockefeller University, New York, USA; 176 Rutgers, The State University of New Jersey, Piscataway, USA; 177 University of Tennessee, Knoxville, USA; 178 Texas A&M University, College Station, USA; 179 Texas Tech University, Lubbock, USA; 180 Vanderbilt University, Nashville, USA; 181 University of Virginia, Charlottesville, USA; 182 Wayne State University, Detroit, USA; 183 University of Wisconsin, Madison, USA

## Abstract

An inclusive search for supersymmetric processes that produce final states with jets and missing transverse energy is performed in pp collisions at a centre-of-mass energy of 8 TeV. The data sample corresponds to an integrated luminosity of 11.7 fb^−1^ collected by the CMS experiment at the LHC. In this search, a dimensionless kinematic variable, *α*_T_, is used to discriminate between events with genuine and misreconstructed missing transverse energy. The search is based on an examination of the number of reconstructed jets per event, the scalar sum of transverse energies of these jets, and the number of these jets identified as originating from bottom quarks. No significant excess of events over the standard model expectation is found. Exclusion limits are set in the parameter space of simplified models, with a special emphasis on both compressed-spectrum scenarios and direct or gluino-induced production of third-generation squarks. For the case of gluino-mediated squark production, gluino masses up to 950–1125 GeV are excluded depending on the assumed model. For the direct pair-production of squarks, masses up to 450 GeV are excluded for a single light first- or second-generation squark, increasing to 600 GeV for bottom squarks.

## Introduction

The standard model (SM) of particle physics has been extremely successful in describing phenomena at the highest energies attained thus far. Nevertheless, it is widely believed to be only an effective approximation of a more complete theory that would supersede the SM at a higher energy scale. Supersymmetry (SUSY) [1–8] is generally regarded as one of the likely extensions to the SM. The theory is based on the unique way to extend the space-time symmetry group underpinning the SM, introducing a relationship between fermions and bosons.

A low-energy realisation of SUSY, e.g. at the TeV scale, is motivated by the cancellation of the quadratically divergent loop corrections to the Higgs boson mass in the SM [7, 8]. In order to avoid a large amount of fine-tuning in these loop corrections, the difference in masses between the top quark and the third-generation squarks must not be too large [9]. While the majority of SUSY particles (sparticles) may be beyond the reach of the Large Hadron Collider (LHC) at the present beam energy and luminosity, the recent discovery of a low-mass Higgs boson candidate [10, 11] motivates “natural” SUSY models in which top and bottom squarks (and gluinos) appear at the TeV scale. For R-parity-conserving SUSY [12], sparticles will be produced in pairs and decay to SM particles and the lightest sparticle (LSP), which is generally assumed to be weakly interacting and massive. Therefore, the pair production of massive coloured sparticles is expected to result in a signature that is rich in jets, in particular those originating from bottom quarks if the third-generation squarks are light, and contains a significant amount of missing transverse energy, , due to the undetected LSPs.

This paper summarises an inclusive search for pair production of massive coloured sparticles in final states with jets and , performed in pp collisions at a centre-of-mass energy $\sqrt{s} = 8\ \mathrm{TeV}$. The analysed data sample corresponds to an integrated luminosity of 11.7±0.5 fb^−1^ [13] collected by the Compact Muon Solenoid (CMS) experiment. Several other searches in this channel have been conducted by both the ATLAS and CMS experiments [14–26]. The strategy of the analysis presented in this paper is based on the kinematic variable *α*_T_, which provides powerful discrimination against multijet production, a manifestation of quantum chromodynamics (QCD), while maintaining sensitivity to a wide range of SUSY models. This analysis extends previous searches based on a similar strategy with samples of pp collisions at $\sqrt{s} = 7\ \mathrm{TeV}$ [24–26].

In order to improve the sensitivity of the analysis to the main production mechanisms of massive coloured sparticles at hadron colliders (squark–squark, squark–gluino, and gluino–gluino), events with significant  and two or more energetic jets are categorised according to the jet multiplicity. Events with two or three reconstructed jets are used to search for squark–squark and squark–gluino production, while events with four or more reconstructed jets probe gluino–gluino production. This classification according to the jet multiplicity is a new feature with respect to the previous analysis [24]. Moreover, to enhance the sensitivity to third-generation squark signatures, events are further categorised according to the number of reconstructed jets identified as originating from bottom quarks (b-quark jets). The analysis also considers a large dynamic range in the scalar sum of the transverse energies of reconstructed jets in order to probe signal models over a large range of mass splittings between the parent sparticle and the LSP, including models characterised by a compressed spectrum [27]. This approach provides sensitivity to a wide variety of SUSY event topologies arising from the pair production and decay of massive coloured sparticles while still maintaining the character of an inclusive search.

## Interpretation with simplified models

To interpret the results of this search, simplified models [28–30] are used. These effective models include only a limited set of sparticles (production and decay) to enable comprehensive studies of individual SUSY event topologies. The result of this search can also be interpreted in a range of other relevant models, such as the constrained minimal supersymmetric extension of the standard model (CMSSM) [31–33] or other effective or complete SUSY models that predict event topologies with two or more energetic jets and significant .

In this paper, we focus on the interpretation in two classes of simplified models, the first of which describes direct pair production of squarks, including top and bottom squarks, that decay to a quark of the same flavour and the LSP. The second class describes gluino-induced production of (off-shell) squarks, again including top and bottom squarks, in which gluino pair production is followed by the decay of each gluino to a quark-antiquark pair and the LSP. The simplified models considered in this analysis are summarised in Table 1. For each model, the LSP is assumed to be the lightest neutralino.

**Table 1:** A summary of the simplified models considered in this analysis, which involve both direct (D) and gluino-induced (G) production of squarks, and their decays. Models D1 and G1 concern the direct or gluino-induced production of first- or second-generation squarks only. Reference models are also defined in terms of the parent (gluino or squark) and LSP sparticle masses

Model	Production/decay mode	Reference model	
		*m*_parent_ [GeV]	*m*_LSP_ [GeV]
D1	$\mathrm{pp}\rightarrow\tilde{\mathrm{q}}\tilde{\mathrm{q}}^{*} \rightarrow\mathrm{q}\tilde{\chi}^{0}_{1}\bar{\mathrm{q}}\tilde{\chi}^{0}_{1}$	600	250
D2	$\mathrm{pp}\rightarrow \tilde{\mathrm{b}}\tilde{\mathrm{b}}^{*} \rightarrow\mathrm{b}\tilde{\chi}^{0}_{1}\bar{\mathrm{b}}\tilde{\chi}^{0}_{1}$	500	150
D3	$\mathrm{pp}\rightarrow \tilde{\mathrm{t}}\tilde{\mathrm{t}}^{*} \rightarrow\mathrm{t}\tilde{\chi}^{0}_{1} \bar{\mathrm{t}}\tilde{\chi}^{0}_{1}$	400	0
G1	$\mathrm{pp}\rightarrow\tilde{\mathrm{g}}\tilde{\mathrm{g}} \rightarrow\mathrm{q}\bar{\mathrm{q}}\tilde{\chi}^{0}_{1} \mathrm{q}\bar{\mathrm{q}}\tilde{\chi}^{0}_{1}$	700	300
G2	$\mathrm{pp}\rightarrow \tilde{\mathrm{g}}\tilde{\mathrm{g}} \rightarrow\mathrm{b}\bar{\mathrm{b}}\tilde{\chi}^{0}_{1} \mathrm{b}\bar{\mathrm{b}}\tilde{\chi}^{0}_{1}$	900	500
G3	$\mathrm{pp}\rightarrow \tilde{\mathrm{g}}\tilde{\mathrm{g}} \rightarrow\mathrm{t}\bar{\mathrm{t}}\tilde{\chi}^{0}_{1} \mathrm{t}\bar{\mathrm{t}}\tilde{\chi}^{0}_{1}$	850	250

Table 1 also defines reference models in terms of the parent (gluino or squark) and LSP sparticle masses, *m*_parent_ and *m*_LSP_, respectively, which are used to illustrate potential yields in the signal region. In the case of the model D3, a massless LSP is considered. The masses are chosen to be reasonably high while still being within the expected sensitivity reach.

## The CMS apparatus

The central feature of the CMS apparatus is a superconducting solenoid of 6 m internal diameter, providing a magnetic field of 3.8 T. Within the superconducting solenoid volume are a silicon pixel and strip tracker, an electromagnetic calorimeter (ECAL) comprising 75 848 lead-tungstate crystals, and a brass/scintillator hadron calorimeter (HCAL). Muons are measured in gas-ionisation detectors embedded in the steel flux return yoke of the magnet. Extensive forward calorimetry complements the coverage provided by the barrel and endcap detectors. The CMS detector is nearly hermetic, which allows for momentum balance measurements in the plane transverse to the beam axis.

CMS uses a right-handed coordinate system, with the origin at the nominal interaction point, the *x* axis pointing to the centre of the LHC ring, the *y* axis pointing up (perpendicular to the plane of the LHC ring), and the *z* axis along the anticlockwise-beam direction. The polar angle *θ* (radians) is measured from the positive *z* axis and the azimuthal angle *ϕ* (radians) is measured in the *x*-*y* plane. Pseudorapidity is defined as *η*=−ln[tan(*θ*/2)].

The silicon pixel and strip tracking systems measure charged particle trajectories with full azimuthal coverage and a pseudorapidity acceptance of |*η*|<2.5. The resolutions on the transverse momentum (*p*_T_) and impact parameter of a charged particle with *p*_T_<40 GeV are typically 1 % and 15 μm, respectively. Muons are measured in the pseudorapidity range |*η*|<2.4. Matching muons to tracks measured in the tracking subdetectors results in a *p*_T_ resolution between 1 and 5 % for *p*_T_≤1 TeV.

The ECAL has an energy resolution of better than 0.5 % for unconverted photons with transverse energies above 100 GeV. The HCAL, when combined with the ECAL, measures jets with a resolution $\Delta E/E \approx 100~\% / \sqrt{E[\mathrm{GeV}]} \oplus 5~\%$. In the region |*η*|<1.74, the HCAL cells have widths of 0.087 in pseudorapidity and 0.087 in azimuth. In the *η*-*ϕ* plane, and for |*η*|<1.48, the HCAL cells map onto 5×5 arrays of ECAL crystals to form calorimeter towers projecting radially outwards from close to the nominal interaction point. At larger values of |*η*|, the size of the towers increases and the matching ECAL arrays contain fewer crystals. Within each tower, the energy deposits in ECAL and HCAL cells are summed to define the calorimeter tower energies, subsequently used to provide the energies and directions of hadronic jets.

The first level (L1) of the CMS trigger system, composed of custom hardware processors, uses information from the calorimeters and muon detectors to select the most interesting events in a fixed time interval of less than 4 μs. The high-level trigger (HLT) processor farm further decreases the event rate, from around 100 kHz to around 300 Hz, before data are stored.

A more detailed description of the CMS detector can be found in Ref. [34].

## Event reconstruction and selection

### Definition of *α*_T_

The *α*_T_ [26, 35] variable is used to reject multijet events efficiently without significant  or with transverse energy mismeasurements, while retaining a large sensitivity to new physics with final-state signatures containing significant .

The measurement of  typically relies on independent sources of information from each of the calorimeter, tracking, and muon subdetectors [36]. Relative to other physics objects, this measurement is particularly sensitive to the beam conditions and detector performance. This difficulty is compounded by the high-energy, high-luminosity hadron collider environment at the LHC and the lack of precise theoretical predictions for the kinematic properties and cross sections of multijet events.

Given these difficulties, the variable *α*_T_ was developed to avoid direct reliance on a measurement of , instead depending solely on the measurements of the transverse energies and (relative) azimuthal angles of jets, which are reconstructed from energy deposits in the calorimeter towers [37]. The variable is intrinsically robust against the presence of jet energy mismeasurements in multijet systems. For dijet events, the *α*_T_ variable is defined as [26, 35]:

1 $$ \alpha_{\mathrm{T}} =\frac{E_{\mathrm{T}}^{\mathrm{j}_2}}{M_{\mathrm{T}}}, $$

where $E_{\mathrm{T}}^{\mathrm{j}_{2}}$ is the transverse energy of the less energetic jet and *M*_T_ is the transverse mass of the dijet system, defined as

2 $$ M_{\mathrm{T}} = \sqrt{ \Biggl( \sum _{i=1}^2 E_{\mathrm{T}}^{\mathrm{j}_i} \Biggr)^2 - \Biggl( \sum_{i=1}^2 p_x^{\mathrm{j}_i} \Biggr)^2 - \Biggl( \sum _{i=1}^2 p_y^{\mathrm{j}_i}, \Biggr)^2}, $$

where $E_{\mathrm{T}}^{\mathrm{j}_{i}}$, $p_{x}^{\mathrm{j}_{i}}$, and $p_{y}^{\mathrm{j}_{i}}$ are, respectively, the transverse energy and *x* or *y* components of the transverse momentum of jet j_*i*_.

For a perfectly measured dijet event with $E_{\mathrm{T}}^{\mathrm{j}_{1}} = E_{\mathrm{T}}^{\mathrm{j}_{2}}$ and jets back-to-back in *ϕ*, and in the limit in which the momentum of each jet is large compared with its mass, the value of *α*_T_ is 0.5. For the case of an imbalance in the measured transverse energies of back-to-back jets, *α*_T_ is reduced to a value smaller than 0.5, which gives the variable its intrinsic robustness with respect to jet energy mismeasurements. A similar behaviour is observed for energetic dijet events that contain neutrinos from the decay of a bottom or charm quark, as the neutrinos are typically collinear with respect to the axis of the heavy-flavour jet. Values significantly greater than 0.5 are observed when the two jets are not back-to-back and are recoiling against significant, genuine .

The definition of the *α*_T_ variable can be generalised for events with two or more jets as follows. The mass scale of the physics processes being probed is characterised by the scalar sum of the transverse energy *E*_T_ of jets considered in the analysis, defined as $H_{\mathrm{T}} = \sum_{i=1}^{n_{\mathrm{jet}}} E_{\mathrm{T}}^{\mathrm{j}_{i}}$, where *n*_jet_ is the number of jets with *E*_T_ above a predefined threshold. The estimator for  is given by the magnitude of the transverse momenta ***p***_**T**_ vectorial sum over these jets, defined as . For events with three or more jets, a pseudo-dijet system is formed by combining the jets in the event into two pseudo-jets. The total *E*_T_ for each of the two pseudo-jets is calculated as the scalar sum of the measured *E*_T_ of the contributing jets. The combination chosen is the one that minimises the absolute *E*_T_ difference between the two pseudo-jets, Δ*H*_T_. This simple clustering criterion provides the best separation between multijet events and events with genuine . Equation (1) can therefore be generalised as: 3


In the presence of jet energy mismeasurements or neutrinos from heavy-flavour quark decays, the direction and magnitude of the apparent missing transverse energy, , and energy imbalance of the pseudo-dijet system, Δ*H*_T_, are highly correlated. This correlation is much weaker for R-parity-conserving SUSY with each of the two decay chains producing the LSP.

### Physics objects

Jets are reconstructed from the energy deposits in the calorimeter towers [37], clustered by the infrared-safe anti-*k*_T_ algorithm [38] with a size parameter of 0.5. In this process, the contribution from each calorimeter tower is assigned a momentum, the absolute value and the direction of which are given by the energy measured in the tower and the position of the tower. The raw jet energy is obtained from the sum of the tower energies and the raw jet momentum by the vectorial sum of the tower momenta, which results in a nonzero jet mass. The raw jet energies are corrected to remove the effects of overlapping pp collisions (pileup) [39, 40] and to establish a relative uniform response of the calorimeter in *η* and a calibrated absolute response in *p*_T_.

The presence of a b-quark jet is inferred by the Combined Secondary Vertex algorithm [41] that incorporates several measurements to build a single discriminating variable that can be used to identify jets originating from bottom quarks with high efficiency and purity. Due to the pixel-detector acceptance, b-quark jets are identified only in the region |*η*|<2.4. In this analysis, the discriminator threshold is chosen such that the probability to misidentify (mistag) jets originating from light-flavour partons (u, d, s quarks or gluons) as b-quark jets is approximately 1 % for jets with transverse momenta of 80 GeV [41]. This threshold results in a b-tagging efficiency, i.e. the probability to correctly identify jets as originating from bottom quarks, in the range 60–70 % [41], dependent on jet *p*_T_.

The reconstruction of photons, electrons and muons is described below. The presence (or absence) of these objects is used to define the event samples for the signal and multiple control regions, the latter of which are used to estimate the background contributions from SM processes in the signal region.

The energy of photons [42] is directly obtained from the ECAL measurement, corrected for zero-suppression and pileup effects. Various identification criteria must be met in order to correctly identify photons with high efficiency and suppress the misidentification of electrons, jets, or spurious ECAL noise as photons. These include the requirements that the shower shape of the energy deposition in the ECAL be consistent with that expected from a photon, the energy detected in the HCAL behind the photon shower must not exceed 5 % of the photon energy, and no matched hits in the pixel tracker must be found. Isolation from other activity in the event is determined through a combination of independent energy sums obtained from each of the HCAL, ECAL, and tracker subdetectors within a cone of $\Delta R = \sqrt{(\Delta\phi)^{2} + (\Delta\eta)^{2}} = 0.3$ around the photon trajectory. These sums are corrected for pileup effects and for the contributions from the photon itself.

The energy of electrons [43] is determined from a combination of the track momentum at the main interaction vertex, the corresponding ECAL cluster energy, and the energy sum of all bremsstrahlung photons attached to the track. Identification criteria similar to those described above for photons are applied, with additional requirements on the associated track that consider the track quality, energy–momentum matching, and compatibility with the main interaction vertex in terms of the transverse and longitudinal impact parameters.

The energy of muons [44] is obtained from the corresponding track momentum, combining measurements from the muon detectors and both the silicon pixel and strip tracking subdetectors. Various track quality criteria are considered when identifying muons, as are the transverse and longitudinal impact parameters with respect to the main interaction vertex.

Isolation of muons and electrons is based on a combination of independent sums from the HCAL, ECAL, and tracker subdetectors and measured relative to the muon or electron transverse momentum. The isolation sums are determined for a cone of radius Δ*R*=0.3 (0.4) around the electron (muon) trajectory and are corrected for the effects of pileup and for the contributions from the lepton itself.

### Event selection for the signal region

Events containing non-collision backgrounds are suppressed by requiring at least one vertex of high-*p*_T_ tracks to be reconstructed in the luminous region. In the case of multiple vertices, the main interaction vertex is defined as the one with the highest scalar sum of $p_{\mathrm{T}}^{2}$ of all associated tracks.

In order to suppress SM processes with genuine  from neutrinos in the final state, events are vetoed if they contain an isolated electron or muon with *p*_T_>10 GeV. Events with an isolated photon with *p*_T_>25 GeV are also vetoed to ensure an all-jet final state.

Jets are required to have transverse energy *E*_T_>50 GeV and |*η*|<3.0. The two highest-*E*_T_ jets must each satisfy *E*_T_>100 GeV. These two *E*_T_ requirements are relaxed for some signal regions, as described below. The highest-*E*_T_ jet is additionally required to satisfy |*η*|<2.5. Events are vetoed that contain rare, spurious signals from the calorimeters [45] that are misidentified as jets. To ensure that the variable  is an unbiased estimator of , events are vetoed if any additional jet satisfies both *E*_T_>50 GeV and |*η*|>3.

Events are required to have *H*_T_>275 GeV to ensure high efficiency for the trigger conditions used to record the event sample, described in Sect. 4.4. The signal region is divided into eight bins in *H*_T_: two bins of width 50 GeV in the range 275<*H*_T_<375 GeV, five bins of width 100 GeV in the range 375<*H*_T_<875 GeV, and a final open bin, *H*_T_>875 GeV. As in Ref. [26], the jet *E*_T_ threshold is scaled down to 37 and 43 GeV for the regions 275<*H*_T_<325 and 325<*H*_T_<375 GeV, respectively. The threshold for the two highest-*E*_T_ jets is also scaled accordingly to 73 and 87 GeV. This is done in order to maintain a background composition similar to that observed for the higher *H*_T_ bins, and also to increase the analysis acceptance for SUSY models characterised by compressed spectra.

Events are further categorised according to the number of jets per event, 2≤*n*_jet_≤3 or *n*_jet_≥4, and the number of reconstructed b-quark jets per event, *n*_b_=0, 1, 2, 3, or ≥4. For the category of events satisfying *n*_jet_≥4 and *n*_b_≥4, the six highest *H*_T_ bins are combined to give a final open bin of *H*_T_>375 GeV.

For events satisfying the selection criteria described above, the multijet background dominates over all other SM backgrounds. As discussed in Sect. 4.1, multijet events populate the region *α*_T_<0.5. The *α*_T_ distribution is characterised by a sharp edge at 0.5, beyond which the multijet event yield falls by several orders of magnitude. Multijet events with extremely rare but large stochastic fluctuations in the calorimetric measurements of jet energies can lead to values of *α*_T_ slightly above 0.5. The edge at 0.5 sharpens with increasing *H*_T_ for multijet events, primarily due to a corresponding increase in the average jet energy and thus an improvement in the jet energy resolution. This effect yields an exponential dependence on *H*_T_ for the ratio of multijet events with a value of *α*_T_ above and below a given threshold value (larger than 0.5), as described further in Sect. 6.

The contribution from multijet events is suppressed by many orders of magnitude by requiring *α*_T_>0.55. As an example, an event that satisfies both *H*_T_=275 (875) GeV and *α*_T_=0.55 must also satisfy  (365) GeV. However, certain classes of rare background events can lead to values of *α*_T_ greater than 0.55, such as those containing beam halo, reconstruction failures, spurious detector noise, or event misreconstruction due to detector inefficiencies. These event classes, with large, non-physical values of , are rejected by applying dedicated vetoes [36], the most important of which are described below.

The first example concerns events containing severe energy mismeasurements as a result of jets being reconstructed within or near to inefficient regions in the ECAL (which amount to ∼1 % of the ECAL channel count) or the instrumentation gap between the ECAL barrel and endcap systems at |*η*|=1.48. These events are identified and vetoed as follows. The negative vector sum of jet transverse momenta when jet *j* is ignored, defined as $-\sum_{i = 1, i \neq j}^{n_{\mathrm{jet}}} \boldsymbol{p}_{\mathbf{T}}^{i}$, is determined for each ignored jet in turn, 1≤*j*<*n*_jet_. An azimuthal distance of Δ*ϕ*<0.5 between the directions of jet *j* and the corresponding vector sum indicates that jet *j* has suffered a sufficiently large energy mismeasurement to satisfy *α*_T_>0.55. The event is rejected if the angular distance in the (*η*,*ϕ*) plane between the affected jet and the closest inefficient ECAL region satisfies Δ*R*<0.3. Similarly, the event is rejected if the *η* position of the affected jet satisfies Δ*η*<0.3 with respect to the ECAL barrel-endcap boundary.

The second example concerns the rare circumstance in which several jets with transverse energies below the *E*_T_ thresholds and aligned in *ϕ* result in significant  relative to the value of  (which is less sensitive to jet *E*_T_ thresholds). This type of background, typical of multijet events, is suppressed while maintaining high efficiency for SM or new physics processes with genuine, significant  by requiring . The measurement of  is provided by the particle-flow (PF) reconstruction framework [46, 47].

**Fig. 1:**
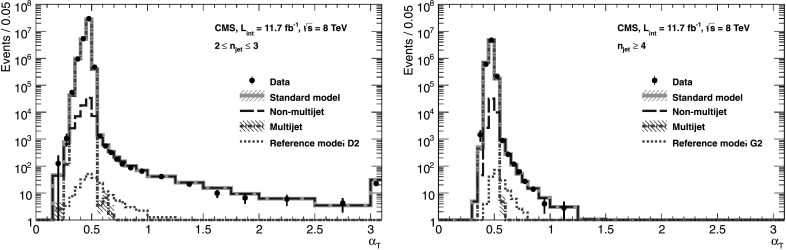
The *α*_T_ distributions of events with *H*_T_>375 GeV that satisfy all the selection criteria described above except the *α*_T_ requirement, categorised according to 2≤*n*_jet_≤3 (*left*) and *n*_jet_≥4 (*right*). An inclusive set of trigger conditions is used to collect the events in data (*black solid circles* with *error bars*). Expected yields as given by simulation are also shown for multijet events (*green dash-dotted line*), non-multijet backgrounds with genuine  as described in Sect. 5 (*blue long-dashed line*), the sum of all aforementioned SM processes (*cyan solid line*) and the reference signal model D2 (*left*, *red dotted line*) or G2 (*right*, *red dotted line*). The statistical uncertainties for the multijet and SM expectations are represented by the hatched areas (visible only for statistically limited bins). The final bin contains all events with *α*_T_>3 (Color figure online)

Figure 1 shows the *α*_T_ distributions of events with *H*_T_>375 GeV that satisfy all the selection criteria described above except the *α*_T_ requirement, categorized according to *n*_jet_. An inclusive set of trigger conditions is used in order to show the full *α*_T_ distribution. The analysis relies on data control samples to estimate the contributions from the multijet and non-multijet backgrounds, as described in Sects. 5 and 6. However, for illustration, the expected yields from simulation of multijet events, non-multijet backgrounds with genuine , the sum of these SM backgrounds, and an example signal model, are also shown in Fig. 1. The expected yield for multijet events that satisfy *α*_T_>0.55, as given by simulation, is less than ten events and is negligible with respect to all other SM backgrounds. Figure 1 highlights the ability of the *α*_T_ variable to discriminate between multijet events and all other SM or new physics processes with genuine  in the final state.

### Trigger conditions

Events are recorded with multiple jet-based trigger conditions, implemented on the HLT computing farm, that require both *H*_T_ and *α*_T_ to lie above predetermined thresholds, as summarised in Table 2. Different trigger conditions are used depending on the analysis *H*_T_ bin. The trigger-level jet energies are corrected to account for scale and pileup effects. The thresholds used in the *H*_T_ binning scheme are shifted up by 25 GeV with respect to the trigger thresholds in order to maintain high efficiency for the *H*_T_ component of the trigger condition.

**Table 2:** Trigger conditions used to record events for each *H*_T_ bin and their efficiencies (with statistical uncertainties) measured in data for each *H*_T_ bin and *n*_jet_ category

Analysis bin	Trigger thresholds		Trigger efficiency [%]	
*H*_T_ [GeV]	*H*_T_ [GeV]	*α*_T_	2≤*n*_jet_≤3	*n*_jet_≥4
275–325	250	0.55	89.1$^{+0.4}_{-0.4}$	83.7$^{+0.6}_{-0.6}$
325–375	300	0.53	98.7$^{+0.2}_{-0.3}$	98.2$^{+0.4}_{-0.5}$
375–475	350	0.52	99.0$^{+0.4}_{-0.5}$	99.7$^{+0.2}_{-0.6}$
≥475	400	0.51	100.0$^{+0.0}_{-0.6}$	100.0$^{+0.0}_{-0.8}$

The trigger efficiency, defined as the probability with which events that satisfy the signal region selection criteria also satisfy the trigger condition, is measured from data for each *n*_jet_ category. The efficiency is measured using a data sample of *μ*+jets events recorded by an independent and unbiased trigger condition that requires an isolated muon satisfying *p*_T_>24 GeV and |*η*|<2.1. The muon is required to be well separated from the nearest jet *j* by requiring Δ*R*(*μ*,*j*)>0.5 and is ignored in the calculation of *H*_T_ and *α*_T_ in order to emulate a genuine  signature.

The measured efficiencies are summarised in Table 2. Non-negligible inefficiencies, which are accounted for in the final result, are observed only for the lowest *H*_T_ bin. The HLT-based trigger conditions are dependent on multiple requirements on quantities determined by the L1 trigger logic, which include combinations of scalar sums of jet *E*_T_ measurements and individual *E*_T_ thresholds on sub-leading jets. The different efficiencies measured for the two *n*_jet_ categories in the lowest *H*_T_ bin are a result of the requirements on L1 trigger quantities that exhibit non-negligible inefficiencies at very low *H*_T_.

## Estimating the non-multijet backgrounds

### Dominant background processes

In the absence of a significant contribution from multijet events, the remaining backgrounds in the signal region stem from SM processes with significant  in the final state.

For events in which no b-quark jets are identified, the largest backgrounds are from the production of W and Z bosons in association with jets. The decay $\mathrm{Z}\to\nu\bar{\nu}$ is the only relevant contribution from Z+jets events. For W+jets events, the two relevant sources are leptonic decays, in which the lepton is not reconstructed or fails the isolation or acceptance requirements, and the decay W→*τν* in which the *τ* decays hadronically and is identified as a jet.

For events satisfying *n*_b_≥1, $\mathrm{t}\bar{\mathrm{t}}$ production followed by semileptonic decays becomes the most important background process. For the subset of events satisfying *n*_b_=1 and 2≤*n*_jet_≤3, the total contribution from the W+jets and Z+jets backgrounds is comparable to the $\mathrm{t}\bar{\mathrm{t}}$ background; otherwise $\mathrm{t}\bar{\mathrm{t}}$ production dominates. Events with three or more reconstructed b-quark jets originate almost exclusively from $\mathrm{t}\bar{\mathrm{t}}$ events, in which one or several jets are misidentified as b-quark jets.

Residual contributions from single-top-quark and diboson production are also expected.

### Definition of the data control samples

Three independent data control samples, binned identically to the signal region, are used to estimate the contributions from the various background processes. These samples are defined by a selection of *μ*+jets, *μμ*+jets, and *γ*+jets events. The event selection criteria for these control samples are defined to ensure that any potential contamination from multijet events is negligible. Furthermore, the selections are also expected to suppress contributions from a wide variety of SUSY models (signal contamination) to a negligible level. The selection criteria that define the three data control samples are chosen such that the composition of background processes and their kinematic properties resemble as closely as possible those of the signal region once the muon, dimuon system, or photon are ignored when computing quantities such as *H*_T_, Δ*H*_T_, , and *α*_T_. This approach emulates the effects, including misreconstruction and acceptance, that lead to the presence of these background processes in the signal region.

The *μ*+jets sample is recorded using a trigger condition that requires an isolated muon satisfying *p*_T_>24 GeV and |*η*|<2.1. The event selection requires exactly one isolated muon that satisfies stringent quality criteria, *p*_T_>30 GeV, and |*η*|<2.1 in order for the trigger to be maximally efficient at (88.0±2.0) %. Furthermore, the transverse mass of the muon and  [46, 47] system must be larger than 30 GeV to ensure a sample rich in W bosons. The muon is required to be separated from the closest jet in the event by the distance Δ*R*>0.5. The event is rejected if two muon candidates are identified that have an invariant mass within a window of ±25 GeV around the mass of the Z boson, regardless of the quality and isolation of the second muon candidate. No selection requirement on *α*_T_ is made in order to increase the statistical precision of the predictions derived from this sample, while the impact of removing the *α*_T_ requirement is tested with a dedicated set of closure tests described in Sect. 5.4.

The *μμ*+jets sample uses the same trigger condition as the *μ*+jets sample. Events are selected by requiring exactly two oppositely charged, isolated muons that satisfy stringent quality criteria and |*η*|<2.1. The highest-*p*_T_ and lowest-*p*_T_ muons must satisfy *p*_T_>30 GeV and *p*_T_>10 GeV, respectively. The invariant mass of the di-muon system is required to be within a window of ±25 GeV around the mass of the Z boson. Both muons are required to be separated from their closest jets in the event by the distance Δ*R*>0.5. Again, no requirement on *α*_T_ is made. These selection criteria lead to a trigger efficiency of 95±2 %, rising to 98±2 % with increasing *H*_T_.

The *γ*+jets sample is selected using a dedicated photon trigger requiring a localised, large energy deposit in the ECAL with *E*_T_>150 GeV that satisfies loose photon identification and isolation criteria [42]. The offline selection requires *H*_T_>375 GeV, *α*_T_>0.55, and a single photon to be reconstructed with *E*_T_>165 GeV, |*η*|<1.45, satisfying tight isolation criteria, and with a minimum distance to any jet of Δ*R*>1.0. For these selection criteria, the photon trigger condition is found to be fully efficient.

### Method

The method used to estimate the non-multijet backgrounds in the signal region relies on the use of transfer factors, which are constructed per data control sample in bins of *H*_T_, *n*_jet_, and *n*_b_. These transfer factors are determined from simulated event samples, which are produced as follows. The production of W and Z bosons in association with jets is simulated with the MadGraph V5 [48] event generator. The production of $\mathrm{t}\bar{\mathrm{t}}$ and single-top quark events is generated with powheg [49], and diboson events are produced with pythia 6.4 [50]. For all simulated samples, pythia 6.4 is used to describe parton showering and hadronisation. The description of the detector response is implemented using the Geant4 [51] package. The simulated samples are normalised using the most accurate cross section calculations currently available, usually with next-to-leading-order (NLO) accuracy. To model the effects of pileup, the simulated events are generated with a nominal distribution of pp interactions per bunch crossing and then reweighted to match the pileup distribution as measured in data.

Each transfer factor is defined as the ratio of expected yields as given by simulation in a given bin of the signal region, $N_{\mathrm{MC}}^{\mathrm{signal}}$, and the corresponding bin of one of the control samples, $N_{\mathrm{MC}}^{\mathrm{control}}$. Each transfer factor is then used to extrapolate from the event yield measured in a data control sample, $N_{\mathrm{obs}}^{\mathrm{control}}$, to an expectation for the event yield in the corresponding bin of the signal region, $N_{\mathrm{pred}}^{\mathrm{signal}}$, via the expression:

4 $$ N_{\mathrm{pred}}^{\mathrm{signal}} = \frac{N_{\mathrm{MC}}^{\mathrm{signal}}}{N_{\mathrm{MC}}^{\mathrm{control}}} \times N_{\mathrm{obs}}^{\mathrm{control}}. $$

Two independent estimates of the irreducible background of $\mathrm{Z}\to \nu\bar{\nu} + \mathrm{jets}$ events are determined from the data control samples comprising Z→*μμ*+jets and *γ*+jets events, both of which have similar kinematic properties when the muons or photon are ignored [52] but different acceptances. Of the *γ*+jets and Z→*μμ*+jets processes, the former has a larger production cross section while the latter has kinematic properties that are more similar to $\mathrm{Z}\to\nu\bar{\nu} + \mathrm{jets}$.

The *μ*+jets data sample provides an estimate for the total contribution from all other SM processes, which is dominated by $\mathrm{t}\bar{\mathrm{t}}$ and W-boson production. Residual contributions from single-top-quark and diboson production are also estimated. For the category of events satisfying *n*_b_≥2, in which the contribution from $\mathrm{Z}\to\nu\bar{\nu} + \text{jets}$ events is suppressed to a negligible level, the *μ*+jets sample is also used to estimate this small contribution rather than using the statistically limited *μμ*+jets and *γ*+jets samples. Hence, only the *μ*+jets sample is used to estimate the total SM background for events satisfying *n*_b_≥2, whereas all three data control samples are used for events satisfying *n*_b_≤1.

In order to maximise sensitivity to potential new physics signatures in final states with multiple b-quark jets, a method that improves the statistical power of the predictions from simulation, particularly for *n*_b_≥2, is employed. The distribution of *n*_b_ is estimated from generator-level information contained in the simulation. The number of reconstruction-level jets matched to underlying bottom quarks ($n_{\mathrm{b}}^{\mathrm{gen}}$), charm quarks ($n_{\mathrm{c}}^{\mathrm{gen}}$), and light-flavoured partons ($n_{\mathrm{q}}^{\mathrm{gen}}$) per event, $N(n_{\mathrm{b}}^{\mathrm{gen}},n_{\mathrm{c}}^{\mathrm{gen}},n_{\mathrm{q}}^{\mathrm{gen}})$, is recorded in bins of *H*_T_ for each *n*_jet_ category. The b-tagging efficiency, *ϵ*, and mistag probabilities, *f*_c_ and *f*_q_, are also determined from simulation for each *H*_T_ bin and *n*_jet_ category, with each quantity averaged over jet *P*_T_ and *η*. Corrections are applied on a jet-by-jet basis to both *ϵ*, *f*_c_, and *f*_q_ in order to match the corresponding measurements from data [41]. This information is sufficient to predict *n*_b_ and thus also determine the event yield *N*(*n*_b_) from simulation for a given *H*_T_ bin and *n*_jet_ category with the expression:

5 $$ N(n_{\mathrm{b}}) = \sum_{n_{\mathrm{jet}}} \sum_{n_{\mathrm{b}}} \bigl(N\bigl(n_{\mathrm{b}}^{\mathrm{gen}}, n_{\mathrm{c}}^{\mathrm{gen}}, n_{\mathrm{q}}^{\mathrm{gen}}\bigr) \times P_{\mathrm{b}} \times P_{\mathrm{c}} \times P_{\mathrm{q}}\bigr), $$

where $n_{\mathrm{b}}^{\mathrm{tag}}$, $n_{\mathrm{c}}^{\mathrm{tag}}$, and $n_{\mathrm{q}}^{\mathrm{tag}}$ are the number of times that a reconstructed b-quark jet is identified as originating from an underlying bottom quark, charm quark, or light-flavoured parton, respectively, and $P_{\mathrm{b}} \equiv P(n_{\mathrm{b}}^{\mathrm{tag}} ; n_{\mathrm{b}}^{\mathrm{gen}}, \epsilon)$, $P_{\mathrm{c}} \equiv P(n_{\mathrm{c}}^{\mathrm{tag}} ; n_{\mathrm{c}}^{\mathrm{gen}}, f_{\mathrm{c}})$, and $P_{\mathrm{q}}\equiv P(n_{\mathrm{q}}^{\mathrm{tag}} ; n_{\mathrm{q}}^{\mathrm{gen}}, f_{\mathrm{q}})$ are the binomial probabilities for this to happen. The outer summation considers all possible combinations of $n_{\mathrm{b}}^{\mathrm{gen}}$, $n_{\mathrm{c}}^{\mathrm{gen}}$, and $n_{\mathrm{q}}^{\mathrm{gen}}$ that satisfy $n_{\mathrm{jet}} = n_{\mathrm{b}}^{\mathrm{gen}} + n_{\mathrm{c}}^{\mathrm{gen}} + n_{\mathrm{q}}^{\mathrm{gen}}$, while the inner summation considers all possible combinations of $n_{\mathrm{b}}^{\mathrm{tag}}$, $n_{\mathrm{c}}^{\mathrm{tag}}$, and $n_{\mathrm{q}}^{\mathrm{tag}}$ that satisfy $n_{\mathrm{b}} = n_{\mathrm{b}}^{\mathrm{tag}} + n_{\mathrm{c}}^{\mathrm{tag}} + n_{\mathrm{q}}^{\mathrm{tag}}$.

The predicted yields are found to be in good statistical agreement with the yields obtained directly from the simulation in the bins with a significant population. The method exploits the ability to make precise measurements of $N(n_{\mathrm{b}}^{\mathrm{gen}}, n_{\mathrm{c}}^{\mathrm{gen}},n_{\mathrm{q}}^{\mathrm{gen}})$, *ϵ*, *f*_c_, and *f*_q_ independently of *n*_b_, which means that event yields for a given b-quark jet multiplicity can be predicted with a higher statistical precision than obtained directly from simulation. Precise measurements of *f*_c_ and *f*_q_ are particularly important for events with *n*_b_≥3, which often occur in the SM because of the presence of mistagged jets in the event. In this case, the largest background is $\mathrm{t}\bar{\mathrm{t}}$, with two correctly tagged b-quark jets and an additional mistagged jet originating from a charm quark or light-flavoured parton.

### Systematic uncertainties on transfer factors

As described in Sect. 5.3, the method to estimate the background contributions from SM processes with significant  is based on an extrapolation from a measurement in a control sample to a yield expectation in the signal region. This approach aims to minimise the sensitivity to simulation mismodelling, as many systematic biases are expected largely to cancel in the ratios used to define the transfer factors. However, a systematic uncertainty is assigned to each transfer factor to account for theoretical uncertainties [52] and residual biases in the simulation modelling of kinematics (e.g. acceptances) and instrumental effects (e.g. reconstruction inefficiencies).

The magnitudes of the systematic uncertainties assigned to the transfer factors are determined from a representative set of closure tests in data. These tests use yields from an event category in one of the three independent data control samples, along with the corresponding transfer factors obtained from simulation, to predict the yields in another event category or data control sample following the prescription defined in Eq. (4). As stated previously, the contamination from multijet events or any potential signal is expected to be negligible. Therefore, the closure tests carried out between control samples probe the properties of the relevant SM non-multijet backgrounds.

**Fig. 2:**
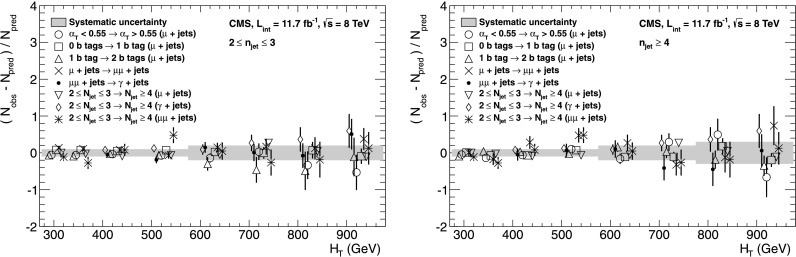
Sets of closure tests that probe for possible *H*_T_-dependent biases associated with the transfer factors obtained from simulation, for the two event categories satisfying 2≤*n*_jet_≤3 (*left*) and *n*_jet_≥4 (*right*). Also shown are *shaded bands* that represent *H*_T_-dependent systematic uncertainties (Color figure online)

Thirteen sets of closure tests are chosen to probe key ingredients of the simulation modelling that may introduce biases in the transfer factors. Each set comprises up to eight independent tests in bins of *H*_T_. Five sets of closure tests are performed independently for each of the two *n*_jet_ categories, and a further three sets are common to both categories, as shown in Fig. 2. For each *n*_jet_ category, the first three sets of closure tests are carried out within the *μ*+jets sample, and probe the modelling of the *α*_T_ distribution in genuine  events (circles), the relative composition between W+jets and top events (squares), and the modelling of the reconstruction of b-quark jets (triangles), respectively. The fourth set (crosses) addresses the modelling of the vector boson samples by connecting the *μ*+jets and *μμ*+jets control samples, with the former sample rich in W+jets events (and also with a significant contribution from top events) and the latter in Z+jets events. The fifth set (solid bullets) deals with the consistency between the Z→*μμ*+jets and *γ*+jets samples, which are both used to provide an estimate of the $\mathrm{Z}\to\nu\bar{\nu} + \text{jets}$ background. Three further sets of closure tests (inverted triangles, diamonds, asterisks), one per data control sample, probe the simulation modelling of the *n*_jet_ distribution.

All sets of closure tests demonstrate, given the statistical precision of each test, that there are no significant biases or dependencies on *H*_T_ exhibited by the transfer factors obtained from simulation. Table 3 summarises the results obtained from constant and linear polynomial fits to each set of closure tests for the two *n*_jet_ categories. The table also lists the best fit values and uncertainties for the constant polynomial fits, which indicate the level of closure averaged across the full *H*_T_ range considered in the analysis. All tests are either statistically compatible with zero bias or at the level of a few percent or less. Finally, Table 3 also summarises the best fit values of the slopes of the linear polynomial fits, which are typically of the order 10^−4^, corresponding to a percent-level change per 100 GeV. However, in all cases, the best fit values are fully compatible with zero, indicating that the level of closure is *H*_T_-independent. The *χ* ^2^ and number of degrees of freedom (dof) of each fit are also quoted and indicate a reasonable goodness-of-fit in all cases except one, which concerns the simulation modelling of the *n*_jet_ distribution in the *μ*+jets sample. The large *χ* ^2^ value is mainly attributable to a single outlier in the bin 675<*H*_T_<775 GeV rather than any significant trend in *H*_T_.

**Table 3:** Results from constant and linear polynomial fits to sets of closure tests performed for each *n*_jet_ category. The symbol identifies the set of closure tests in Fig. 2. The final four rows probe the simulation modelling of the *n*_jet_ distribution. The ^†^ indicates the fit repeated with a single outlier removed

*n*_jet_	Symbol	Set of closure tests	Constant polynomial fit		Linear polynomial fit	
			Constant	*χ* ^2^/dof	Slope [10^−4^ GeV^−1^]	*χ* ^2^/dof
2–3	◯	*α*_T_<0.55→*α*_T_>0.55 (*μ*+jets)	−0.06±0.02	2.43/7	−1.3±2.2	2.10/6
2–3	□	0 b tags→1 b tag (*μ*+jets)	0.07±0.02	1.49/7	−1.6±1.6	0.54/6
2–3	△	1 b tag→2 b tags (*μ*+jets)	−0.07±0.03	4.19/7	−2.7±3.0	3.41/6
2–3	×	*μ*+jets→*μμ*+jets	0.10±0.03	5.64/7	−1.1±2.3	5.40/6
2–3	•	*μμ*+jets→*γ*+jets	−0.06±0.04	5.93/5	4.2±4.3	4.98/4
≥4	◯	*α*_T_<0.55→*α*_T_>0.55 (*μ*+jets)	−0.05±0.03	9.58/7	3.0±2.9	8.47/6
≥4	□	0 b tags→1 b tag (*μ*+jets)	−0.03±0.03	5.88/7	−1.0±1.9	5.59/6
≥4	△	1 b tag→2 b tags (*μ*+jets)	−0.02±0.03	7.35/7	1.1±2.2	7.08/6
≥4	×	*μ*+jets→*μμ*+jets	0.08±0.07	12.9/7	4.8±4.3	11.7/6
≥4	•	*μμ*+jets→*γ*+jets	−0.03±0.10	2.85/5	−4.0±7.0	2.52/4
≥2	▽	2≤*n*_jet_≤3→*n*_jet_≥4 (*μ*+jets)	−0.03±0.02	17.3/7	0.0±1.0	17.3/6
≥2^†^	▽	2≤*n*_jet_≤3→*n*_jet_≥4 (*μ*+jets)	−0.04±0.01	6.10/6	−1.4±1.1	4.46/5
≥2	◊	2≤*n*_jet_≤3→*n*_jet_≥4 (*γ*+jets)	0.12±0.05	2.42/5	6.0±4.7	0.77/4
≥2	∗	2≤*n*_jet_≤3→*n*_jet_≥4 (*μμ*+jets)	−0.04±0.07	9.76/7	4.9±4.4	8.51/6

Once it is established that no significant bias or trend is observed for any set of closure tests, uncorrelated systematic uncertainties on the transfer factors are determined for five independent regions in *H*_T_: 275–325, 325–375, 375–575, 575–775, and ≥775 GeV. Conservative estimates for the systematic uncertainties are based on the variance in the level of closure for all individual tests, weighted according to the statistical uncertainties associated with each test, within a given *H*_T_ region. This procedure yields estimates of 10 % (10 %), 10 % (10 %), 10 % (10 %), 20 % (20 %), and 20 % (30 %) for the five *H*_T_ regions defined above for events satisfying 2≤*n*_jet_≤3 (*n*_jet_≥4), as indicated by the shaded bands in Fig. 2.

The effect on the transfer factors of uncertainties related to the modelling of b-quark jets in simulation is studied in detail. After correcting the b-tagging efficiency and mistag probability determined in simulation for residual differences as measured in data, the corresponding uncertainties on these corrections are propagated to the transfer factors. In addition, several robustness tests are performed, e.g. treating c-quark jets as b-quark jets. While the absolute yields ($N_{\mathrm{MC}}^{\mathrm{signal}}$ and $N_{\mathrm{MC}}^{\mathrm{control}}$) are susceptible to systematic biases, the transfer factors are not, because changes to $N_{\mathrm{MC}}^{\mathrm{signal}}$ and $N_{\mathrm{MC}}^{\mathrm{control}}$ are strongly correlated. The relative change in the transfer factors is found to be negligible, at the sub-percent level. Hence, the aforementioned *H*_T_-dependent systematic uncertainties are also used for each *n*_b_ category and are treated as uncorrelated among *n*_b_ categories.

## Estimating the multijet background

The contribution from multijet events is expected to be negligible, at or below the percent-level relative to the yields expected from non-multijet backgrounds, even for the most inclusive definition of the signal region, defined by *H*_T_>275 GeV, *α*_T_>0.55, and no requirement on *n*_jet_ or *n*_b_. The expected yield is further suppressed to ≪1 event with the application of more stringent thresholds on any of the variables *H*_T_, *n*_jet_, or *n*_b_.

Any potential contamination from multijet events via the effects described in Sects. 4.1 and 4.3 can be estimated by exploiting the *H*_T_ dependence of the ratio of events with a value of *α*_T_ above and below some threshold, $R_{\alpha_{\mathrm{T}}}(H_{\mathrm{T}})$. This dependence on *H*_T_ is modelled as a falling exponential function, $R_{\alpha_{\mathrm{T}}}(H_{\mathrm{T}}) = A\mathrm{e}^{-k H_{\mathrm{T}}}$ [26], where the parameters *A* and *k* are the normalisation and decay constant parameters, respectively. The exponential model is validated in a multijet-enriched data sideband, defined by the event selection criteria for the signal region, described in Sect. 4.3, but with the requirement . A measurement of the decay constant *k* is made in a further multijet-enriched sample defined by the event selection criteria for the signal region but with the requirement *α*_T_<0.55.

The estimate of the multijet contamination in the signal region for a given *H*_T_ bin is determined from the product of the ratio $R_{\alpha_{\mathrm{T}}}$, as given by the exponential model, and the yield in a data control sample defined by the event selection for the signal region but with the requirement *α*_T_<0.55. This event sample is recorded with a set of trigger conditions that require only *H*_T_ to be above the same thresholds as used by the signal region triggers listed in Table 2.

Further details on the exponential model and its use in the likelihood model are found in Sect. 7.

## Confronting data with the SM-only hypothesis

For a given category of events satisfying requirements on both *n*_jet_ and *n*_b_, a likelihood model of the observations in multiple data samples is used to obtain a consistent prediction of the SM backgrounds and to test for the presence of a variety of signal models. It is written as:

6 $$\begin{aligned} &{L_{n_{\mathrm{jet}},n_{\mathrm{b}}} = L_{\mathrm{SR}} \times L_{\mu} \times L_{\mu\mu} \times L_{\gamma},\quad (0 \leq n_{\mathrm{b}} \leq 1)} \end{aligned}$$

7 $$\begin{aligned} &{L_{n_{\mathrm{jet}},n_{\mathrm{b}}} = L_{\mathrm{SR}} \times L_{\mu},\quad (n_{\mathrm{b}} \geq 2)} \end{aligned}$$

where *L*_SR_ describes the yields in the eight *H*_T_ bins of the signal region where exactly *n*_jet_ jets and *n*_b_ b-quark jets are required. In each bin of *H*_T_, the observation is modelled as a Poisson-distributed variable about the sum of the SM expectation and a potential signal contribution (assumed to be zero in the following discussion), where the SM expectation is the sum of the multijet and non-multijet components. The non-multijet component is related to the expected yields in the *μ*+jets, *μμ*+jets, and *γ*+jets control samples via the transfer factors derived from simulation, as described in Sect. 5.3. The likelihood functions *L*_*μ*_, *L*_*μμ*_, and *L*_*γ*_ describe the yields in the *H*_T_ bins of the *μ*+jets, *μμ*+jets, and *γ*+jets control samples in the same category of *n*_jet_ and *n*_b_ as the signal region. For the category of events satisfying *n*_b_≥2, only the *μ*+jets control sample is used in the likelihood to determine the total contribution from all non-multijet SM backgrounds in the signal region. The estimate of the contribution from multijet events in a given *H*_T_ bin of the signal region relies on the exponential model $R_{\alpha_{\mathrm{T}}}(H_{\mathrm{T}}) = A\mathrm{e}^{-k H_{\mathrm{T}}}$, as described in Sect. 6. The systematic uncertainties (10–30 %) associated with the transfer factors, discussed in Sect. 5.4, are accommodated in the likelihood function by a nuisance parameter per transfer factor. The measurements of these parameters are assumed to follow a log-normal distribution.

In order to test the compatibility of the observed yields with the expectations from only SM processes, the likelihood function is maximised over all fit parameters. For each of the eight categories of events defined by requirements on *n*_jet_ and *n*_b_, the goodness-of-fit of the SM-only hypothesis is determined by considering simultaneously up to eight bins in *H*_T_ from the signal region and up to 22 bins from the three control samples. No significant tension is observed in the signal and control regions, which are well described by the SM hypothesis. The p-values obtained are found to be uniformly distributed, with a minimum observed value of 0.1. Table 4 summarises the observed yields and fit results in bins of *H*_T_ for events in the signal region categorised according to *n*_jet_ and *n*_b_.

**Table 4:** Event yields observed in data and fit results with their associated uncertainties in bins of *H*_T_ for events in the signal region that are categorised according to *n*_jet_ and *n*_b_. The final *H*_T_>375 GeV bin is inclusive for the *n*_jet_≥4 and *n*_b_≥4 category

	*n*_jet_	*n*_b_	*H*_T_ bin [GeV]							
			275–325	325–375	375–475	475–575	575–675	675–775	775–875	875–∞
SM	2–3	0	$6235^{+100}_{-67}$	$2900^{+60}_{-54}$	$1955^{+34}_{-39}$	$558^{+14}_{-15}$	$186^{+11}_{-10}$	$51.3^{+3.4}_{-3.8}$	$21.2^{+2.3}_{-2.2}$	$16.1^{+1.7}_{-1.7}$
Data	2–3	0	6232	2904	1965	552	177	58	16	25
SM	2–3	1	$1162^{+37}_{-29}$	$481^{+18}_{-19}$	$341^{+15}_{-16}$	$86.7^{+4.2}_{-5.6}$	$24.8^{+2.8}_{-2.7}$	$7.2^{+1.1}_{-1.0}$	$3.3^{+0.7}_{-0.7}$	$2.1^{+0.5}_{-0.5}$
Data	2–3	1	1164	473	329	95	23	8	4	1
SM	2–3	2	$224^{+15}_{-14}$	$98.2^{+8.4}_{-6.4}$	$59.0^{+5.2}_{-6.0}$	$12.8^{+1.6}_{-1.6}$	$3.0^{+0.9}_{-0.7}$	$0.5^{+0.2}_{-0.2}$	$0.1^{+0.1}_{-0.1}$	$0.1^{+0.1}_{-0.1}$
Data	2–3	2	222	107	58	12	5	1	0	0
SM	≥4	0	$1010^{+34}_{-24}$	$447^{+19}_{-16}$	$390^{+19}_{-15}$	$250^{+12}_{-11}$	$111^{+9}_{-7}$	$53.3^{+4.3}_{-4.3}$	$18.5^{+2.4}_{-2.4}$	$19.4^{+2.5}_{-2.7}$
Data	≥4	0	1009	452	375	274	113	56	16	27
SM	≥4	1	$521^{+25}_{-17}$	$232^{+15}_{-12}$	$188^{+12}_{-11}$	$106^{+6}_{-6}$	$42.1^{+4.1}_{-4.4}$	$17.9^{+2.2}_{-2.0}$	$9.8^{+1.5}_{-1.4}$	$6.8^{+1.2}_{-1.1}$
Data	≥4	1	515	236	204	92	51	13	13	6
SM	≥4	2	$208^{+17}_{-9}$	$103^{+9}_{-7}$	$85.9^{+7.2}_{-6.9}$	$51.7^{+4.6}_{-4.7}$	$19.9^{+3.4}_{-3.0}$	$6.8^{+1.2}_{-1.3}$	$1.7^{+0.7}_{-0.4}$	$1.3^{+0.4}_{-0.3}$
Data	≥4	2	204	107	84	59	24	5	1	2
SM	≥4	3	$25.3^{+5.0}_{-4.2}$	$11.7^{+1.7}_{-1.8}$	$6.7^{+1.4}_{-1.2}$	$3.9^{+0.8}_{-0.8}$	$2.3^{+0.6}_{-0.6}$	$1.2^{+0.3}_{-0.4}$	$0.3^{+0.2}_{-0.1}$	$0.1^{+0.1}_{-0.1}$
Data	≥4	3	25	13	4	2	2	3	0	0
SM	≥4	≥4	$0.9^{+0.4}_{-0.7}$	$0.3^{+0.2}_{-0.2}$	$0.6^{+0.3}_{-0.3}$	–	–	–	–	–
Data	≥4	≥4	1	0	2	–	–	–	–	–

**Fig. 3:**
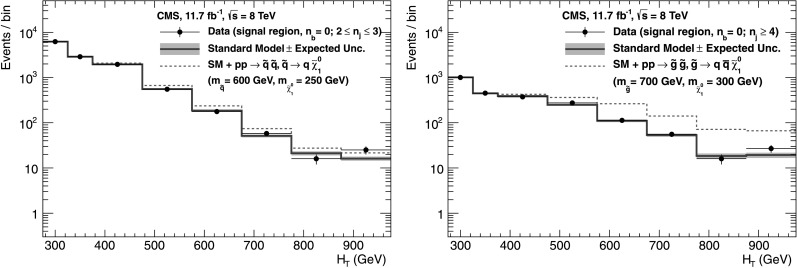
Event yields observed in data (*solid circles*) and SM expectations with their associated uncertainties (*solid lines* with *bands*) in bins of *H*_T_ for the signal region when requiring exactly zero b-quark jets and 2≤*n*_jet_≤3 (*left*) or *n*_jet_≥4 (*right*). For illustration only, the expectations for the reference mass points of the signal models D1 (*left*, *red dashed line*) and G1 (*right*, *red dashed line*) are superimposed on the SM-only expectations (Color figure online)

**Fig. 4:**
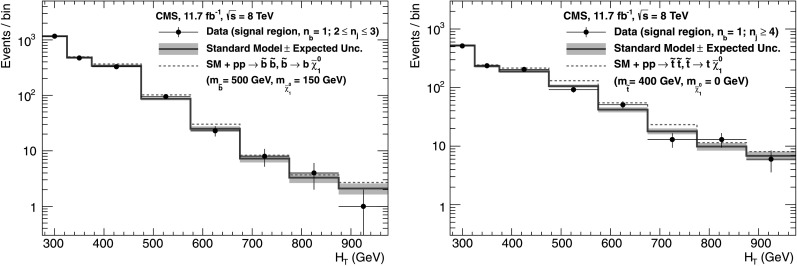
As for Fig. 3, but requiring exactly one b-quark jet and 2≤*n*_jet_≤3 (*left*) or *n*_jet_≥4 (*right*). Example signal yields are for the reference mass points of the signal models D2 (*left*, *red dashed line*) and D3 (*right*, *red dashed line*) (Color figure online)

**Fig. 5:**
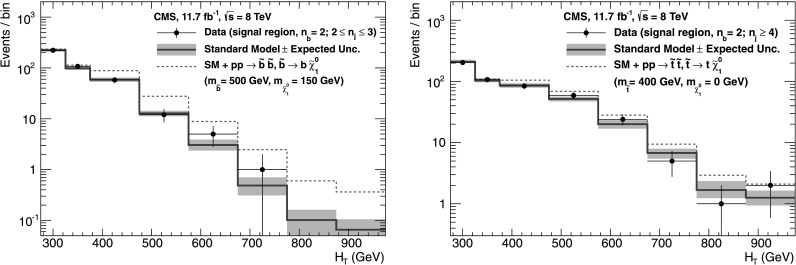
As for Fig. 3, but requiring exactly two b-quark jets and 2≤*n*_jet_≤3 (*left*) or *n*_jet_≥4 (*right*). Example signal yields are for the reference mass points of the signal models D2 (*left*, *red dashed line*) and D3 (*right*, *red dashed line*) (Color figure online)

**Fig. 6:**
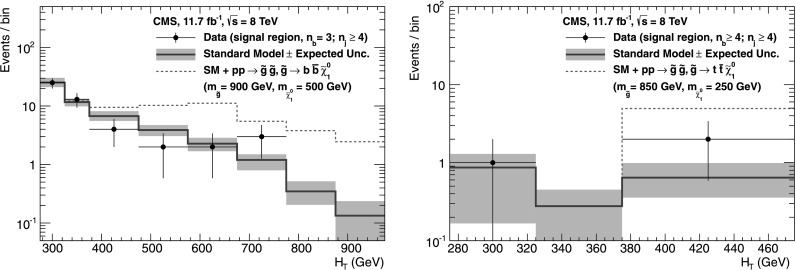
As for Fig. 3, but requiring *n*_jet_≥4 and exactly three (*left*) or at least four (*right*) b-quark jets. Example signal yields are for the reference mass points of the signal models G2 (*left*, *red dashed line*) and G3 (*right*, *red dashed line*) (Color figure online)

**Fig. 7:**
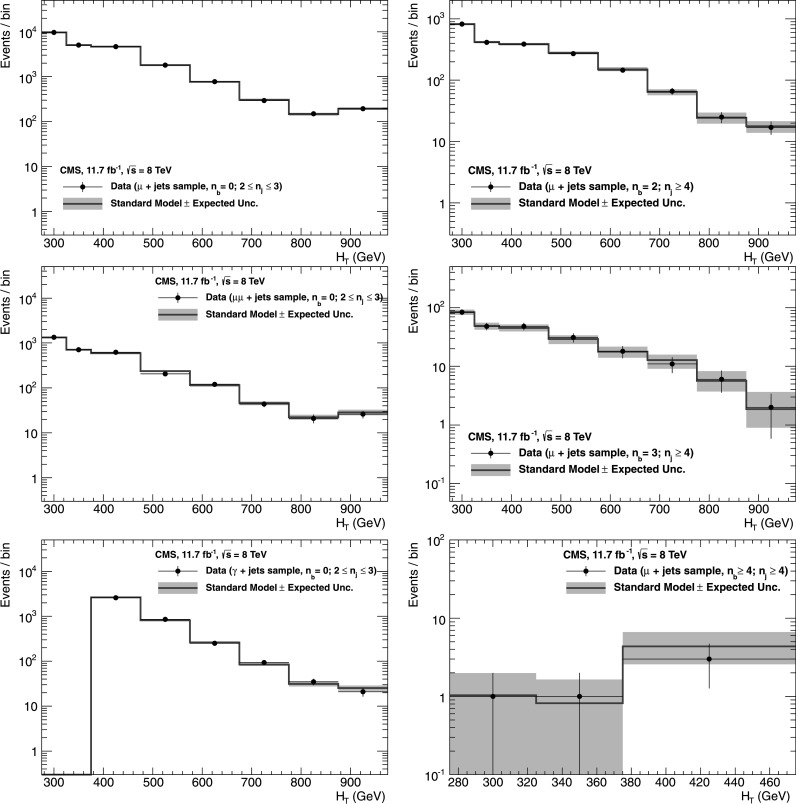
Event yields observed in data (*solid circles*) and SM expectations with their associated uncertainties (*solid lines* with *bands*) in bins of *H*_T_ for: the *μ*+jets (*top left*), *μμ*+jets (*middle left*), and *γ*+jets (*bottom left*) control samples when requiring 2≤*n*_jet_≤3 and exactly zero b-quark jets; and the *μ*+jets control sample when requiring *n*_jet_≥4 and exactly two (*top right*), three (*middle right*), or at least four (*bottom right*) b-quark jets (Color figure online)

Comparisons of the observed yields and the SM expectations in bins of *H*_T_ for events categorised according to *n*_jet_ and containing exactly zero, one, or two b-quark jets are shown in Figs. 3, 4, and 5, respectively. Similarly, Fig. 6 shows the *H*_T_-binned observed yields and SM expectations for events satisfying *n*_jet_≥4 and *n*_b_=3 (left) or *n*_b_≥4 (right). For illustration, Figs. 3–6 include the expected yields from various reference models, as defined in Table 1. Figure 7 (left column) shows the observed yields and SM expectations in the *H*_T_ bins of the *μ*+jets, *μμ*+jets, and *γ*+jets control samples for events satisfying 2≤*n*_jet_≤3 and *n*_b_=0. Figure 7 (right column) shows the observed yields and SM expectations in the *H*_T_ bins of the *μ*+jets sample for events satisfying *n*_jet_≥4 and *n*_b_=2, *n*_b_=3, or *n*_b_≥4.

The maximum-likelihood values for the decay constant and normalisation parameters, *k* and *A*, of the exponential model for the multijet background are obtained independently for each of the eight event categories. The value of the nuisance parameter *k* is constrained via a measurement in a multijet-enriched data sideband, as described in Sect. 6. No constraint is applied to the normalisation term. In the nominal fit, the maximum-likelihood value of the normalisation parameter for each event category is found to be compatible with zero within uncertainties. Furthermore, the expected yields obtained from an alternate fit, in which the normalisation parameters are fixed to zero, agree well with those obtained from the nominal fit.

## Interpretation of the results

Limits are set in the parent sparticle and LSP mass parameter space of the simplified models listed in Table 1. The CL_S_ method [53, 54] is used to compute the limits, with the one-sided (LHC-style) profile likelihood ratio as the test statistic [55]. The sampling distributions for the test statistic are built by generating pseudo-data from the likelihood function, using the respective maximum-likelihood values of the nuisance parameters under the SM background-only and signal-plus-background hypotheses. Signal contributions in each of the data samples are considered, though the only significant contribution occurs in the signal region and not the control samples. Table 5 specifies the event categories, defined in terms of *n*_jet_ and *n*_b_, used to provide interpretations in the different simplified models.

**Table 5:** A summary of the event categories used to provide an interpretation in the various simplified models considered in this paper

Model	*n*_jet_	*n*_b_
D1	2–3	0
D2	2–3	1, 2
D3	≥4	1, 2
G1	≥4	0
G2	≥4	2, 3, ≥4
G3	≥4	2, 3, ≥4

Event samples for the simplified models are generated at leading order with pythia 6.4 [50]. Inclusive, process-dependent, NLO calculations of SUSY production cross sections, with next-to-leading-logarithmic (NLL) corrections, are obtained with the program prospino [56–61]. The samples are generated using the CTEQ6L1 [62] PDFs. The distribution of the number of pp interactions per bunch crossing for the simulated samples matches that observed in data.

Various experimental uncertainties on the expected signal yield are considered for each interpretation. Signal acceptance in the kinematic region defined by 0<*m*_parent_−*m*_LSP_<175 GeV or *m*_parent_<300 GeV is due in part to the presence of initial-state radiation. Given the large associated uncertainties from simulation for this kinematic region, no interpretation is provided. Otherwise, the experimental systematic uncertainties are determined for each point in the mass parameter space of each simplified model. Models are categorised according to the mass splitting between the parent sparticle and the LSP, with those satisfying 175<*m*_parent_−*m*_LSP_<350 GeV deemed to be characterised by a compressed spectrum. For a given category of model, i.e. with a compressed spectrum or otherwise (as defined above), the systematic uncertainties are relatively stable throughout the mass plane, thus a single conservative value is considered for each category.

Estimates of the various systematic uncertainties for models with a compressed spectrum or otherwise are summarised in Tables 6 and 7, respectively. Contributions from the analysis selection are dominated by uncertainties on the PDFs, jet energy scale (JES), and modelling of the efficiency and mistag probability of b-quark jets in simulation. The total systematic uncertainties provided in the tables also account for the uncertainty of 4.4 % on the luminosity measurement [13] and contributions from other event selection criteria, such as: the trigger conditions; the removal of events containing isolated muons, electrons, or photons; and filters to suppress classes of rare, pathological events, as described in Sect. 4.3. Each of these individual contributions is below 4 %. The total systematic uncertainty on the expected signal yield for the various simplified models is found to be in the range 12–23 % and is accounted for with a nuisance parameter, the measurement of which is assumed to follow a lognormal distribution.

**Table 6:** Estimates of the dominant systematic uncertainties (%), defined in the text, on the analysis efficiency for various simplified models that are characterised by a small mass splitting (i.e. compressed spectrum) between the parent sparticle and LSP. The totals also reflect contributions from additional systematic uncertainties described in the text. The region *m*_parent_−*m*_LSP_<350 GeV is kinematically forbidden for the G3 model

Model	D1	D2	D3	G1	G2	G3
PDF	10.0	10.0	10.0	10.0	10.0	–
JES	4.1	4.8	6.5	5.6	7.3	–
b-tagging	2.4	2.2	0.8	3.1	2.7	–
Total	12.9	13.1	13.9	13.9	14.5	–

**Table 7:** Estimates of the dominant systematic uncertainties (%), defined in the text, on the analysis efficiency for various simplified models that are characterised by a large mass splitting between the parent sparticle and LSP. The totals also reflect contributions from additional systematic uncertainties described in the text

Model	D1	D2	D3	G1	G2	G3
PDF	10.0	10.0	10.0	10.0	10.0	10.0
JES	1.1	0.9	3.5	0.8	1.5	0.5
b-tagging	5.8	2.7	1.6	6.6	10.1	19.4
Total	13.4	12.3	12.9	14.0	16.0	23.0

**Fig. 8:**
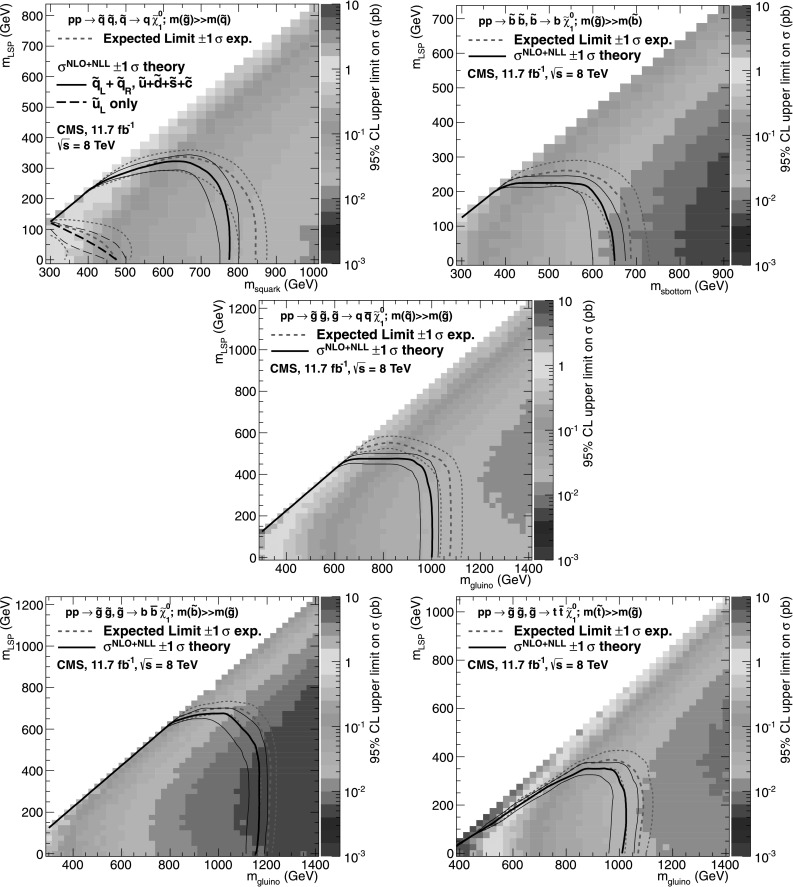
Observed upper limit on the production cross section at 95 % CL (indicated by the *colour scale*) as a function of the parent and LSP sparticle masses for simplified models involving: the direct pair production of eight first- and second-generation squarks with degenerate masses or only a single light squark (D1, *top left*); the direct pair production of bottom squarks (D2, *top right*); and pair-produced gluinos followed by the decay of each gluino to the LSP and pairs of first- and second-generation quarks (G1, *middle*), bottom quarks (G2, *bottom left*), or top quarks (G3, *bottom right*). The *black solid* (or *dashed*) *thick line* indicates the observed exclusion assuming NLO+NLL SUSY production cross section. The *black solid* (or *dashed*) *thin lines* represent the observed exclusions when varying the cross section by its theoretical uncertainty. The *purple dashed thick* (*thin*) *line* indicates the median (±1*σ*) expected exclusion. No interpretation is provided for the kinematic region defined by 0<*m*_parent_−*m*_LSP_<175 GeV or *m*_parent_<300 GeV, as discussed in the text (Color figure online)

Figure 8 shows the observed upper limit on the production cross section at 95 % confidence level (CL) as a function of the parent sparticle and LSP masses for various simplified models. The point-to-point fluctuations are due to the finite number of pseudo-experiments used to determine the observed upper limit. The observed excluded regions are determined with NLO+NLL cross sections for squark pair production assuming decoupled gluinos (and vice versa), i.e. the decoupled sparticle has a sufficiently high mass such that it does not contribute significantly to the cross section. Also shown are the observed excluded regions when varying the production cross section by its theoretical uncertainty, and the expected excluded region with the ±1 standard-deviation variations.

Two sets of excluded regions are provided for the model D1, as shown in Fig. 8 (top left). The larger of the two excluded regions is determined assuming an eightfold degeneracy for the masses of the first- and second-generation squarks, $\tilde{\mathrm{q}}_{\mathrm{L}}$ and $\tilde{\mathrm{q}}_{\mathrm{R}}$ ($\tilde{\mathrm{q}} = \tilde{\mathrm{u}}$, $\tilde{\mathrm{d}}$, $\tilde{\mathrm{s}}$, and $\tilde{\mathrm{c}}$), and decoupled third-generation squarks and gluinos. The smaller of the two excluded regions assumes the pair production of a single light squark, ũ_L_, with the gluino and all other squarks decoupled to high masses. The models D2 and D3 assume the pair production of a single bottom and top squark, respectively.

Table 8 lists the expected signal yields and analysis efficiencies in the region *H*_T_>375 GeV for each of the reference models defined in Table 1. The yields and efficiencies are summed over the individual event categories used for each interpretation, as listed in Table 5. The observed and expected upper limits (95 % CL) on the cross section are also quoted, which can be compared with the NLO+NLL SUSY production cross section and its theoretical uncertainty.

**Table 8:** Summary of expected yields, analysis efficiencies, and upper limits for the reference models defined in Table 1 using the event categories defined in Table 5. The first row specifies the reference model. The second and third rows quote the expected yield and analysis efficiency (with statistical uncertainties) for the region *H*_T_>375 GeV. The fourth row quotes the NLO+NLL SUSY production cross section (with theoretical uncertainty). For the model D1, this cross section assumes an eightfold mass degeneracy. In the case of only a single light squark, the cross section is 25±4 fb. The fifth and sixth rows quote the observed and expected upper limits (95 % CL) on the production cross section

Reference model	D1	D2	D3	G1	G2	G3
Expected yield	358.3±8.9	78.1±2.4	90.6±2.4	416±13	52.0±1.7	25.3±0.7
Efficiency [%]	16.0±0.4	10.2±0.3	2.9±0.1	10.4±0.3	9.4±0.3	2.9±0.1
Theoretical cross section [fb]	196±35	86±13	357±51	434±81	60±14	97±21
Observed upper limit [fb]	113.2	42.3	360.8	103.0	15.0	46.2
Expected upper limit [fb]	103.1	31.2	240.6	65.2	12.3	35.3

The estimates of mass limits are determined from the observed exclusion based on the theoretical production cross section, less one-standard-deviation uncertainty. The most stringent mass limit on the parent sparticle, $m_{\mathrm{parent}}^{\mathrm{best}}$, is generally obtained at low LSP masses. Generally speaking, the excluded mass range for *m*_parent_ is bounded from below by the kinematic region considered for each model, yielding an exclusion that is generally valid for the region $m_{\mathrm{LSP}} + 175\ \mathrm{GeV} \lesssim m_{\mathrm{parent}} \lesssim m_{\mathrm{parent}}^{\mathrm{best}}$. Whether an exclusion can be determined for very small mass splittings, satisfying *m*_parent_−*m*_LSP_<175 GeV, requires further detailed studies of the modelling of, for example, initial-state radiation, JES, or the identification of b-quark jets. The upper bound on *m*_parent_ weakens for increasing values of LSP mass until a value $m_{\mathrm{LSP}}^{\mathrm{best}}$ is reached, beyond which no exclusion on *m*_parent_ can be set.

Table 9 summarises the most stringent observed and expected mass limits, in terms of $m_{\mathrm{parent}}^{\mathrm{best}}$ and $m_{\mathrm{LSP}}^{\mathrm{best}}$, obtained for the simplified models considered in this paper. The observed exclusion for each simplified model is generally weaker than expected at the level of 1–2 standard deviations. This feature is attributed to the small upward fluctuations in data in either the region *H*_T_>875 GeV for the *n*_b_=0 category or 475<*H*_T_<675 GeV for the categories of events satisfying 1≤*n*_b_≤2. Candidate events in these regions have been examined and do not exhibit any non-physical behaviour. The expected search sensitivity has improved with respect to the analysis based on the $\sqrt{s} = 7\ \mathrm{TeV}$ dataset [24] by as much as 225 and 150 GeV for $m_{\mathrm{parent}}^{\mathrm{best}}$ and $m_{\mathrm{LSP}}^{\mathrm{best}}$, respectively.

**Table 9:** Summary of the mass limits obtained for various simplified models. The limits indicate the observed (expected) search sensitivity for each simplified model, where $m_{\mathrm{parent}}^{\mathrm{best}}$ and $m_{\mathrm{LSP}}^{\mathrm{best}}$ represent the largest mass beyond which no limit can be set for squarks or gluinos and the LSP, respectively. Limits are quoted for the model D1 assuming both an eightfold mass degeneracy ($\tilde{\mathrm{q}}$) and only a single light squark (ũ_L_). No exclusion is observed in the mass parameter space considered for the model D3

Model	D1 ($\tilde{\mathrm{q}}$)	D1 (ũ_L_)	D2	D3	G1	G2	G3
$m_{\mathrm{parent}}^{\mathrm{best}}$ [GeV]	750 (850)	450 (475)	600 (675)	– (520)	950 (1050)	1125 (1200)	950 (1075)
$m_{\mathrm{LSP}}^{\mathrm{best}}$ [GeV]	300 (325)	100 (125)	200 (250)	– (100)	450 (550)	650 (700)	325 (375)

**Fig. 9:**
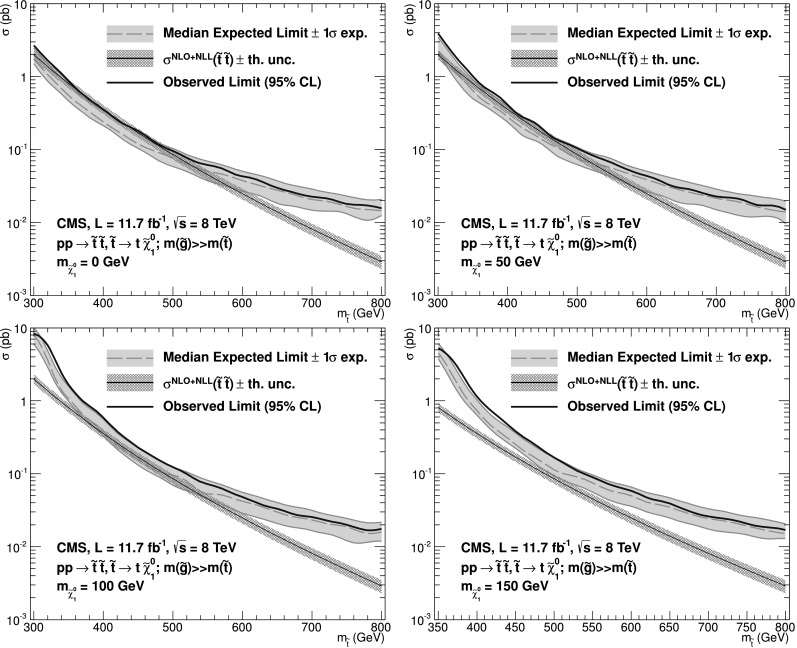
Excluded cross sections versus top-squark mass $m_{\tilde{\mathrm{t}}}$ for the model D3, in which pair-produced top squarks each decay to a top quark and the LSP with a mass *m*_LSP_=0 (*top left*), 50 (*top right*), 100 (*bottom left*), and 150 GeV (*bottom right*). The observed upper limit (95 % CL) on the production cross section is shown as a function of $m_{\tilde{t}}$ (*solid line*), along with the expected upper limit and ±1*σ* experimental uncertainties (*long-dashed line* with *shaded band*), and the NLO+NLL top-squark pair production cross section and theoretical uncertainties (*dotted line* with *shaded band*) (Color figure online)

Figure 9 shows the observed upper limit at 95 % CL on the production cross section as a function of the top-squark mass ($m_{\tilde{\mathrm{t}}}$) for the model D3 when considering different LSP masses in the range 0–150 GeV. No exclusion on possible top-squark masses is observed when considering the theoretical production cross section, less 1*σ* uncertainty. However, the expected exclusion covers the ranges 300–520, 320–520, and 420–480 GeV for *m*_LSP_=0, 50, and 100 GeV, respectively. No exclusion is expected for the LSP with a mass greater than 100 GeV. The expected reach for the D3 model is summarised in Table 9.

## Summary

An inclusive search for supersymmetry with the CMS experiment is reported, based on a data sample of pp collisions collected at $\sqrt{s} = 8\ \mathrm{TeV}$, corresponding to an integrated luminosity of 11.7±0.5 fb^−1^. Final states with two or more energetic jets and significant , as expected from the production and decay of massive squarks and gluinos, have been analysed.

The analysis strategy is to maximise the sensitivity of the search to a wide variety of SUSY event topologies arising from squark–squark, squark–gluino, and gluino–gluino production and decay, particularly those with third-generation squark signatures, while still maintaining the inclusive nature of the search. The signal region is binned according to the number of reconstructed jets, the scalar sum of the transverse energy of jets, and the number of jets identified to originate from bottom quarks. The sum of standard model backgrounds per bin has been estimated from a simultaneous binned likelihood fit to event yields in the signal region and *μ*+jets, *μμ*+jets, and *γ*+jets control samples. The observed yields in the signal region are found to be in agreement with the expected contributions from standard model processes. Limits are set in the SUSY particle mass parameter space of simplified models, with an emphasis on the different production mechanisms of coloured SUSY particles, third-generation squark signatures, and compressed-spectrum scenarios. The results can also be used to perform interpretations in other relevant models, such as the CMSSM.

In the context of simplified models, gluino masses below ∼1 TeV are excluded at the 95 % CL under the assumptions that gluinos are produced in pairs and each decays to a quark–antiquark pair and a light LSP via an off-shell squark. The mass limit varies in the range 950–1125 GeV depending on the squark flavour. The most constraining mass limits on the LSP from the decay of a gluino are in the range 325–650 GeV depending on the decay mode. For the direct production of first- and second-generation squark pairs, each of which is assumed to decay to a quark of the same flavour and the LSP, masses below 750 GeV are excluded (95 % CL) under the assumption of an eightfold mass-degeneracy. The most constraining mass limit on the LSP is 300 GeV. These limits weaken to 450 and 100 GeV respectively if only a single squark is assumed to be light. For the direct production of bottom squark pairs, each of which is assumed to decay to a bottom quark and the LSP, masses below 600 GeV are excluded. No exclusion is possible for an LSP mass beyond 200 GeV. No exclusion is observed for the direct pair production of top squarks, each of which is assumed to decay to a top quark and the LSP. However, an exclusion is expected for top squark masses as high as ∼500 GeV and an LSP mass as high as 100 GeV. The limits on the LSP masses are also generally valid for compressed-spectrum models with mass splittings between the parent sparticle and LSP as low as ∼200 GeV.

The analysis strategy reported here, in conjunction with the increase in centre-of-mass energy to 8 TeV, has increased the coverage of the SUSY parameter space with respect to previous searches. However, a large range of the SUSY parameter space still remains to be probed by the LHC.
